# An Electro-Magnetic Log (EML) Integrated Navigation Algorithm Based on Hidden Markov Model (HMM) and Cross-Noise Linear Kalman Filter

**DOI:** 10.3390/s25041015

**Published:** 2025-02-08

**Authors:** Haosu Zhang, Liang Yang, Lei Zhang, Yong Du, Chaoqi Chen, Wei Mu, Lingji Xu

**Affiliations:** 1 Southern Marine Science and Engineering Guangdong Laboratory (Zhuhai), Zhuhai 519000, China; zhanghs7@mail.sysu.edu.cn (H.Z.); yangliang7@mail2.sysu.edu.cn (L.Y.); chenchq26@mail2.sysu.edu.cn (C.C.); muwei@sml-zhuhai.cn (W.M.); 2 School of Ocean Engineering and Technology, Sun Yat-Sen University, Zhuhai 519000, China; 3 State Power Investment Group (Zhuhai Hengqin) Thermoelectric Co., Ltd., Zhuhai 519000, China; zhanglei30@spic.com.cn; 4 Wuhan National Laboratory for Optoelectronics, Huazhong Institute of Electro-Optics, Wuhan 430223, China; duyong_duli@163.com

**Keywords:** inertial navigation system, Kalman filter (KF)-integrated navigation, AUV, ocean current estimation

## Abstract

In this paper, an EML (electro-magnetic log) integrated navigation algorithm based on the HMM (hidden Markov model) and CNLKF (cross-noise linear Kalman filter) is proposed, which is suitable for SINS (strapdown inertial navigation system)/EML/GNSS (global navigation satellite system) integrated navigation systems for small or medium-sized AUV (autonomous underwater vehicle). The algorithm employs the following five techniques: ① the HMM-based pre-processing algorithm of EML data; ② the CNLKF-based fusion algorithm of SINS/EML information; ③ the MALKF (modified adaptive linear Kalman filter)-based algorithm of GNSS-based calibration; ④ the estimation algorithm of the current speed based on output from MALKF and GNSS; ⑤ the feedback correction of LKF (linear Kalman filter). The principle analysis of the algorithm, the modeling process, and the flow chart of the algorithm are given in this paper. The sea trial of a small-sized AUV shows that the endpoint positioning error of the proposed/traditional algorithm by this paper is 20.5 m/712.1 m. The speed of the water current could be relatively accurately estimated by the proposed algorithm. Therefore, the algorithm has the advantages of high accuracy, strong anti-interference ability (it can effectively shield the outliers of EML and GNSS), strong adaptability to complex environments, and high engineering practicality. In addition, compared with the traditional DVL (Doppler velocity log), EML has the advantages of great concealment, low cost, light weight, small size, and low power consumption.

## 1. Introduction

The continuous utilization and depletion of terrestrial resources have led people to turn their attention to the exploitation of the ocean, which contains a series of resources needed for production and life, providing a solution to the urgent need for resource shortage. Along with the growing demand for the exploitation of marine resources, more advanced and smarter marine equipment has become an indispensable tool for competition in the marine field. In the past two decades, AUV has been proven to be efficient in performing complex underwater tasks. AUV integrates technologies including hydro-acoustic communication, intelligent control, sensors, and others. It has the advantages of good autonomy, good flexibility, and a wide range of activities, and it plays an important strategic role in the fields of ocean exploration and underwater rescue. The AUV’s ability to accomplish tasks is limited by the underwater navigation system, which is the core technology for AUV development [1,2,3]. Different from navigation technology on land, underwater navigation needs to solve the difficult problems of long working time, harsh working environment, high concealment, and so on.

The most commonly and earliest navigation method applied to AUV is dead reckoning (DR), whose core principle is to determine its own position by integrating the AUV’s velocity in real time. The disadvantage of this method is low accuracy. In addition to the DR method, AUVs can also employ an inertial navigation system (INS) for pure inertial calculation, which is based on Newton’s third law and uses an inertial measurement unit (IMU) to measure the angular velocity of the AUV’s rotation around its three axes and the linear acceleration along three axes. The measured acceleration is integrated over time to obtain velocity in three directions, and then the velocity and position vectors of the AUV are derived from data measured by IMU. The most commonly used navigation equipment is the strapdown inertial navigation system (SINS), which consists of a three-axis gyro, a three-axis accelerometer, a calculation circuit board, internal brackets, and a shell. The SINS is fixed directly on the carrier. Due to the measurement noises of the gyro and accelerometer in the SINS, the positioning error gradually increases over time. Therefore, for practical application, underwater velocimetry equipped for integrated navigation is required [1,2,3]. Underwater velocimetry devices include a Doppler velocity log (DVL), acoustic correlation log (ACL), differential pressure sensor speedometer (DPSS), tune odometry (ODO), electro-magnetic log (EML), etc. The advantages of DVL and ACL are high accuracy of measurement and the ability to measure the 3D velocity vector of AUV relative to the ground. The combination of SINS/DVL is currently the mainstream underwater navigation method. The achievements of its research are very fruitful, e.g., the Doppler water track aided navigation [4], the analysis of navigation key techniques [5], a coupled method based on improved dual adaptive factors [6], an algorithm using the velocity filter [7], the performance for sensor selection [8], hardware design [9], the gain of compensation adaptive filtering [10], a method based on the improved adaptive filter [11], a hybrid approach based on an improved AR (auto regressive) model and MAA (motion attitude assist) [12], an algorithm using an indirect feedback Kalman [13], and a tightly coupled method using the regeneration of partial DVL measurements [14]. In addition to DVL, other devices can also be added to the SINS/DVL-integrated system to form a tightly coupled system, e.g., the SINS/DVL/PS (pressure sensor) method based on RIMM (robust interacting multiple models) [15], SINS/DVL/PS navigation with limited DVL measurements [16].

Many scholars have proposed various improvement algorithms for such a mainstream navigation system, such as the fusion method with a cross-correlated process and measurement noise [17], optimal Kalman fusion with cross-correlated sensor noises [18], and a calibration method based on particle swarm optimization [19]. Among these improvement algorithms, adaptive filter (AF) is a very important type. Besides the previous papers [10,11], adaptive H-infinite KF [20] was also proposed. Such a navigation system often employs USBL (ultra-short baseline, acoustic positioning system) [21] for the calibration or estimation of current speed [22]. A GNSS (global navigation satellite system) is also commonly used for the calibration of SINS in a robot or vehicle [23].

These are many research findings of studies on SINS/DVL-integrated navigation systems. An advantage of DVL and ACL is high precision. However, they also have many disadvantages, including high price, large size, heavy weight, complicated installation, outliers in measurement output, etc. In order to solve the shortcomings of DVL, several new velocity measuring devices have been proposed as follows: DSPP is a low-cost instrument that can measure the velocity reference to water of the AUV based on Bernoulli’s principle and can be used for underwater integrated navigation [24,25]. The RMP (revolutions per minute) measured by the PT (propeller tachometer) or ODO can also be used for underwater integrated navigation [26,27]. The EML is a device that works base on the Hall effect to measure forward velocity through water [28,29,30]. These three devices have the common advantages of good concealment (no signals such as acoustic waves are emitted outward), low price, small size, light weight, easy installation, and so on, among which EML is the most accurate and widely used. However, the current SINS/EML algorithm has a great scope for improvement in terms of accuracy and anti-interference capability.

Due to the measurement principle, DPSS, PT/ODO, and EML can only measure velocity through water. In contrast, the integrated navigation requires velocity over ground, and there is a difference between these two speeds in the presence of water currents. Therefore, the high-accuracy SINS/EML-integrated navigation algorithm must consider water currents. Additionally, while the AUV is sailing in the intermediate water layer, the acoustic waves of DVL or ACL cannot detect the bottom of the water. In this case, the water currents should also be taken into account. There are lots of studies on current estimation and compensation. In addition to the above literature [22], there are many other methods, including a model-based high-gain observer [31], a high-gain observer for current estimation [32], localization in a field with a spatiotemporally varying water current [33], and current compensation using an RBF neural network [34].

Besides these studies, there are also many other advanced algorithms that can be used for underwater integrated navigation, including a multi-sensor fusion by the augmentation of the RBF neural network [35], an adaptive navigation algorithm with deep learning [36], an EKF (extended Kalman filter) for an autonomous underwater glider [37], and a UKF (unscented Kalman filter) [38,39].

For solving the problems of current mainstream navigation systems with low stealth (emitting acoustic waves outward) and high cost, the EML is introduced in this paper to take the place of the DVL or ACL. In order to overcome the disadvantages of EML, such as the inability to measure the velocity to the ground, the existence of outliers, and the low accuracy of navigation combined with SINS, the algorithm in this paper employed the following five techniques: ① pre-processing of EML data with HMM- ② SINS/EML-integrated navigation based on CNLKF; ③ calibration on the water surface based on MALKF and GNSS’s output as a measurement; ④ current estimation on the water surface with MALKF and GNSS’s output; ⑤ utilization of the feedback correction method for improving the accuracy of the LKF model. The sea trial of small-sized AUVs shows that the endpoint positioning error rate of this algorithm is 0.134%, and the positioning error is 20.5 m at endpoint, while the error rate of the traditional algorithm is 4.64% and the positioning error is 712.1 m. In the calculation of the error rate, the accurate voyaging distance is estimated by longitude and latitude measurements from GNSS. The maximum error of eastward (northward) current estimation of this algorithm is less than 0.14 m/s (0.095 m/s) with the currents measured by drifting buoys as benchmarks. Compared with the commonly used DVL (Doppler velocity log), EML has the advantages of high concealment, low cost, light weight, small size, and low power consumption. Therefore, the algorithm has the advantages of high accuracy, strong anti-interference ability (it can effectively shield the outliers of EML and GNSS), strong adaptability to adapt complex environment, and high engineering practicality.

The theoretical framework and process of the algorithm are given in Section 2 of this paper, and results of the trial are given in Section 3. Then, in Section 4, the conclusion of the paper and prospects of following developments are given.

## 2. Algorithm

**Figure 1:**
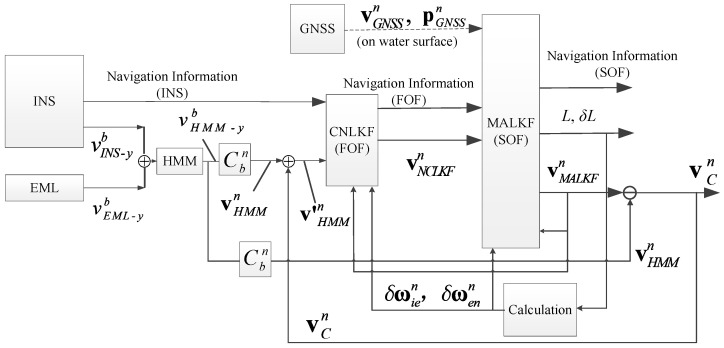
A schematic diagram of the current estimation and integrated navigation algorithm based on HMM.

The basic principle of the algorithm is shown in Figure 1. For the sake of simplicity, SINS is abbreviated as INS. The definitions of INS, EML, HMM, CNLKF, MALKF, and GNSS in Figure 1 were explained earlier. FOF and SOF in Figure 1 denote first-order filter and second-order filter, respectively. The four frames chosen in this paper are defined as follows:

① *b*-frame: the carrier-frame, right-front-upper frame of carrier or IMU strapped to carrier. The origin of the frame is the rotation center or gravity center of the carrier. The rotation center is the center point of the carrier when it is turning. Three axes are noted as *x*, *y*, and *z*, respectively.
② *n*-frame: the orthogonal frame aligned with East-North-Up (ENU) geodetic axes. The origin of the frame is the rotation center or gravity center of the carrier. The three axes are noted as *E*, *N*, and *U*, respectively.
③ *e*-frame: the earth-centered earth-fixed (ECEF) orthogonal frame. The origin of the coordinate is the center of earth or the rotation center of the carrier.
④ *i*-frame: the earth-centered fixed orthogonal frame, which is non-rotating during the alignment and navigation stages.

The $v_{INS-y}^{b}$, $v_{EML-y}^{b}$, $v_{HMM-y}^{b}$, $v_{HMM}^{n}$, and $v_{C}^{n}$ in Figure 1 are velocity along the *y*-axis (forward velocity) calculated by INS, the forward velocity measured by EML, the forward velocity from EML after HMM filtering, the velocity vector of HMM in the *n*-frame (obtained by the velocity vector in the *b*-frame and attitude transformation matrix), and the estimated current vector in the *n*-frame, respectively. $C_{b}^{n}$ is the attitude transformation matrix from the *b*-frame to the *n*-frame. $C_{b}^{n}$ and $v_{HMM}^{n}$ are specified in the following Equations (5) and (21), respectively. $v_{CNLKF}^{n}$, $v_{MALKF}^{n}$, and ${v^{\prime }}_{HMM}^{n}$ are the velocity vector from CNLKF, the vector output by MALKF, and the compensated vector from HMM (current compensation is performed with the relationship ${v^{\prime }}_{HMM}^{n}=v_{HMM}^{n}+v_{C}^{n}$), respectively. The horizontal and depth channels of the AUV are decoupled, and the depth channel is generally damped by the depth gauge (DG). The upward component of $v_{C}^{n}$ is generally small. Thus, only the components in the horizontal plane in ${v^{\prime }}_{HMM}^{n}=v_{HMM}^{n}+v_{C}^{n}$ should be considered. $v_{GNSS}^{n}$ and $p_{GNSS}^{n}$ are the velocity vector and position vector in the *n*-frame obtained from GNSS (generally calculated by $v_{GNSS-y}^{b}$ from GNSS and $C_{b}^{n}$). These two vectors are only available when the AUV is on the water surface (indicated by dashed lines in Figure 1). L and δL are latitude and latitude errors. $\omega_{ie}^{n}$ and $\omega_{en}^{n}$ are the earth’s rotation angular velocity vector and the navigation system’s rotation angular velocity vector in the *n*-frame. $\delta \omega_{ie}^{n}$ and $\delta \omega_{en}^{n}$ are the calculated errors of $\omega_{ie}^{n}$ and $\omega_{en}^{n}$, respectively, which are specified in Equation (33) below. The navigation information in Figure 1 refers to parameters that need to be output except for velocities. It should be noted that $v_{EML-y}^{b}$, $v_{HMM-y}^{b}$, and $v_{HMM}^{n}$ are all velocities through water; $v_{INS-y}^{b}$, $v_{CNLKF}^{n}$, $v_{MALKF}^{n}$, $v_{GNSS}^{n}$, and ${v^{\prime }}_{HMM}^{n}$ are all velocities over ground. In the following, the calculation process is described in detail.

The velocities from INS and EML are fused by HMM to obtain the velocity ($v_{HMM-y}^{b}$) through water with effectively suppressed noise. ${v^{\prime }}_{HMM}^{n}$ can be obtained from $v_{HMM-y}^{b}$, $C_{b}^{n}$, $v_{HMM}^{n}$, and $v_{C}^{n}$. By combining ${v^{\prime }}_{HMM}^{n}$ and other navigation parameters, the CNLKF (FOF) model can be established, from which the velocity vector $v_{CNLKF}^{n}$ and other navigation information (position, acceleration, attitude angle, attitude angular velocity, etc.) can be obtained. Until the first calibration by GNSS, $v_{C}^{n}$ is unknown, and we can assume that it is 0 or an empirical value. If the AUV could not receive information from GNSS, the $v_{CNLKF}^{n}$ from CNLKF (FOF) and other navigation information are used as the final outputs. When the AUV emerges from underwater and receives GNSS signals, the navigation information from CNLKF (FOF) is used to establish the MALKF (SOF) model, and the measurement of GNSS is used to perform the recursive operation of MALKF to obtain $v_{MALKF}^{n}$ and other navigation parameters as the final outputs. Additionally, the estimated $v_{C}^{n}$ is obtained by subtracting $v_{HMM}^{n}$ from $v_{MALKF}^{n}$. Superimposing $v_{C}^{n}$ with $v_{HMM}^{n}$ to obtain ${v^{\prime }}_{HMM}^{n}$ can effectively improve the accuracy of the integrated navigation.

This current velocity is used for compensation until the next calibration by GNSS. Since variation of current speed is generally not drastic, such correction can ensure a certain level of accuracy of integrated navigation. In addition, to further improve the navigation accuracy, the algorithm employs the following feedback correction technique: the velocity over ground and errors $\delta \omega_{ib}^{n}$ and $\delta \omega_{en}^{n}$ calculated based on latitude *L* are fed back into a specific force equation and LKF model. The frequency of GNSS-based calibration depends on the period of current variation. Constant or slowly varying currents require calibration at long intervals, while currents with a short varying period of change require frequent calibrations.

The inputs of the algorithm include the measurement data from EML, INS, and GNSS, while the outputs consist of navigation information (such as the position, velocity, attitude angles, and angular velocities of the platform) and the estimated ocean current. The algorithm incorporates three filters: HMM, CNLKF, and MALKF. The y-axis velocity of the platform, as measured by EML and INS, serves as the input to the HMM. After a coordinate transformation, the velocity output from the HMM is combined with the estimated ocean current fed back from the MALKF to form the input to the CNLKF. Additionally, the navigation information from the INS is also input to the CNLKF. The outputs of the CNLKF, together with the navigation information from the INS and the GNSS measurement data, are then fed into the MALKF. The MALKF outputs the navigation information estimated by the proposed algorithm. Finally, by subtracting the HMM velocity output (after coordinate transformation) from the MALKF velocity output, the 3D ocean current vector can be estimated. In summary, the output of CNLKF (FOF) will be taken as final outputs when $v_{GNSS}^{n}$ and $p_{GNSS}^{n}$ are not received. In contrast, when $v_{GNSS}^{n}$ and $p_{GNSS}^{n}$ are received, the MALKF (SOF) is activated, and its output is taken as the final output.

The proposed algorithm introduces a filtering framework that, although comprising three filters, functions as an integrated system. Each filter is designed to handle the type of data it is most suited to the process, ensuring high accuracy and computational efficiency. The HMM is responsible for preprocessing the measurement data from the EML. The CNLKF integrates data from the HMM and the INS, while the MALKF combines data from the CNLKF and the GNSS, estimates the ocean current, and feeds back model parameters to improve the accuracy of the CNLKF. If a single filter were used to process and integrate all the data simultaneously, it would inevitably result in low computational speed, reduced processing efficiency, and decreased accuracy. The EML has advantages such as low cost, small size, and low power consumption. However, its limitations, including high measurement noise and frequent outliers, restrict its broader application. This study proposes utilizing the HMM to preprocess EML data, reducing its measurement noise, eliminating outliers, and improving the quality of the EML data. Consequently, this preprocessing enhances the accuracy of integrated navigation using EML and INS.

### 2.1. Working Principle of EML

**Figure 2:**
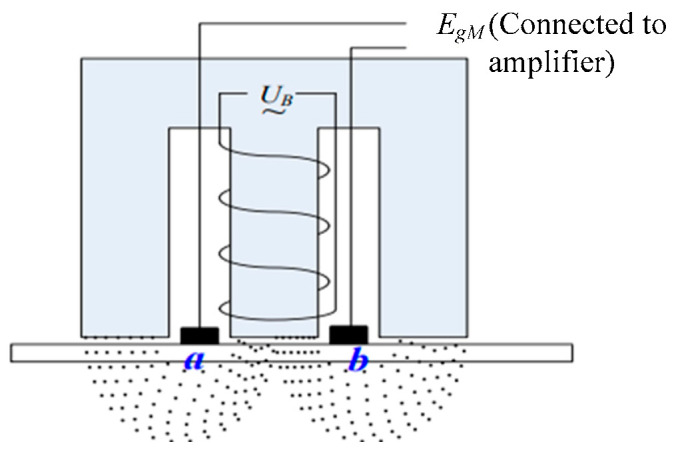
Schematic diagram of the working principle of the EML.

When the AUV is sailing, the water flows through the EML in the direction opposite to the AUV’s movement. Then, the water flows between the two electrodes *a* and *b* (As shown in Figure 2), cutting magnetic induction lines and causing the two electrodes to generate an induced electromotive force, as shown as follows in Equations (1a) and (1b):

(1a) $$E_{gM}=\frac{d\Phi_{B}}{dt}=B\times l\times \left(v_{y}^{b}-v_{C-y}^{b}\right)$$

(1b) $$v_{y}^{b}-v_{C-y}^{b}=\frac{1}{B\times l}\times E_{gM}$$

where $E_{gM}$ denotes the magnitude of the induced electromotive force, *Φ_B_* denotes the magnitude of the magnetic flux, *B* is the strength of the magnetic field, and *l* is the height of the cross section of the magnetic field. In addition, $v_{y}^{b}$ and $v_{C-y}^{b}$ denote the forward velocity of the AUV (the *y* component represents the forward direction) and the *y*-axis component of the current velocity vector, respectively. If there is no current, $v_{y}^{b}$ can be calculated by dividing the induced electromotive force by *B* × *l*. However, due to the presence of current in the real environment, the EML cannot measure velocity over ground.

### 2.2. HMM Algorithm

The error of EML measurement does not accumulate over time, but the measurement is noisy and contains outliers. The velocity calculated from the INS is less noisy, and it contains few outliers, but the error accumulates over time (because of the zero drift of the gyro and accelerometer). In this paper, the above two velocities were fused based on the HMM filter. The error of velocity from the HMM does not accumulate over time, and the noise can be effectively suppressed. Based on this fusion, most of the outliers can be effectively removed and replaced. The expressions of these velocities are as follows:

(2a) $$\left\{\begin{array}{l} v_{EML}^{b}=v^{b}-v_{C}^{b}+W_{EML}^{b}\\ v_{INS}^{b}=v^{b}+W_{INS}^{b}\\ v_{HMM}^{b}=v^{b}-v_{C}^{b}+W_{HMM}^{b}\approx v^{b}-v_{C}^{b} \end{array}\right.$$

In Equation (2a), $v_{EML}^{b}$, $v_{INS}^{b}$, $v_{HMM}^{b}$, $v_{C}^{b}$, and $v^{b}$ are the velocity vector from the EML (obtained by expanding the forward velocity measured by the EML into a velocity vector), the velocity vector solved by the INS and the vector from the HMM (obtained by the relationship $v_{HMM}^{b}={\left[\begin{array}{ccc} 0 & v_{HMM-y}^{b} & 0 \end{array}\right]}^{T}$), the vector (generally unknown and can be obtained based on methods like calibration by GNSS, measurement of current, a priori information of the current, etc.) of current speed and the real velocity vector (generally unknown and it can be estimated by $v_{HMM}^{b}$ and $v_{C}^{b}$) of the AUV in the *b*-frame, respectively. $W_{EML}^{b}$, $W_{INS}^{b}$, and $W_{HMM}^{b}$ are noises of EML, INS, and HMM, respectively. They are given by the correlation: $W_{HMM}^{b}\approx W_{INS}^{b}<W_{EML}^{b}$. Since the EML can only measure the forward velocity, which is critical to the accuracy of integrated navigation, it is the focus of this study. The expressions for forward velocity are shown as follows in Equation (2b):

(2b) $$\left\{\begin{array}{l} v_{EML-y}^{b}=v_{y}^{b}-v_{C-y}^{b}+w_{EML-y}^{b}\\ v_{INS-y}^{b}=v_{y}^{b}+w_{INS-y}^{b}\\ v_{HMM-y}^{b}=v_{y}^{b}-v_{C-y}^{b}+w_{HMM-y}^{b}\approx v_{y}^{b}-v_{C-y}^{b} \end{array}\right.$$

where $v_{EML-y}^{b}$, $v_{INS-y}^{b}$, $v_{HMM-y}^{b}$, $v_{C-y}^{b}$, and $v_{y}^{b}$ are the y-axis components of $v_{EML}^{b}$, $v_{INS}^{b}$, $v_{HMM}^{b}$, $v_{C}^{b}$, and $v^{b}$, respectively.

The principle of the HMM algorithm is shown in Equation (2a). $v_{EML-y}^{b}$ is measured by the EML. $v_{INS-y}^{b}$ is derived by the INS. $v_{HMM-y}^{b}$ is obtained from the HMM. $v_{C-y}^{b}$ is generally unknown and can be obtained by calibration based on the GNSS, measurements of currents, etc. $v_{C-y}^{b}$ is unknown, and it can be estimated from $v_{HMM-y}^{b}$ and $v_{EML-y}^{b}$. The model of the HMM is shown as follows in Equation (3):

(3) $$\left\{\begin{array}{l} X_{k+1}=F_{k,k-1}\cdot X_{k}+W_{k}\\ Z_{k}=H_{k}\cdot X_{k}+V_{k} \end{array}\right.$$

where $W_{k}$ and $V_{k}$ are system noise and measurement noise, respectively. $X_{k}$ and $Z_{k}$ are state vector and measurement vector, respectively, and their components are $X_{k}=\left[\begin{array}{c} v_{EML-y}^{b}\\ v_{INS-y}^{b} \end{array}\right]$ and $Z_{k}=\left[v_{INS-y}^{b}\right]$. $F_{k,k-1}$ and $H_{k}$ are state transition matrix and measurement matrix, respectively. Assuming that the elements composing these two matrices are $F_{ij}$ and $H_{ij}$, respectively, the following correlations are obtained: $\sum_{i=1}^{2}\sum_{j=1}^{2}F_{ij}=1$,$\sum_{i=1}^{1}\sum_{j=1}^{2}H_{ij}=1$ and $F_{ij},\;H_{ij}>0$. The specific values of $F_{ij}$ and $H_{ij}$ are chosen empirically. For EML and INS of common accuracy, the recommended values are: $F_{k,k-1}=\left[\begin{array}{cc} 0.4 & 0.1\\ 0.1 & 0.4 \end{array}\right]$, $H_{k}=\left[\begin{array}{cc} 0.5 & 0.5 \end{array}\right]$. The initial values of $W_{k}$ and $V_{k}$ are set by the technical index of inertial sensors in the INS and the measurement accuracy of the EML.

The recursive algorithm of the HMM model is similar to that of KF, as shown in the following equation:

(4) $$\left\{\begin{array}{l} {\hat{X}}_{k,k-1}=F_{k,k-1}{\hat{X}}_{k-1}\\ {\hat{X}}_{k}={\hat{X}}_{k,k-1}+K_{off}(Z_{k}-H_{k}{\hat{X}}_{k,k-1})\\ P_{k-1}^{-1}=P_{k-1,k-2}^{-1}+H_{k-1}^{T}R_{k-1}^{-1}H_{k-1}\\ P_{k,k-1}=F_{k,k-1}P_{k-1}F_{k,k-1}^{T}+Q_{k-1}\\ K_{off}=P_{k,k-1}H_{k}^{T}{\left(H_{k}P_{k,k-1}H_{k}^{T}+R_{k}\right)}^{-1} \end{array}\right.$$

where ${\hat{X}}_{k,k-1}$ is the one-step prediction state variable; $K_{off}$ is the gain matrix; $R_{k-1}$ is the measurement noise matrix; $P_{k,k-1}$ and $P_{k-1,k-2}$ are both one-step prediction variance matrices; $P_{k-1}$ is the variance matrix; $Q_{k-1}$ is the system noise matrix. The above process and algorithm can be used to obtain $v_{HMM-y}^{b}$ or $v_{HMM}^{b}$.

### 2.3. Pure Inertial Solution Algorithm

The pure inertial solution algorithm is basic knowledge in the field of inertial navigation. Hence, it is not elaborated on here. Next, we introduce the attitude angles used in this article. The attitude angle is defined as:

(5) $$\begin{array}{l} C_{b}^{n}=\left[\begin{array}{ccc} \cos\psi & -\sin\psi & 0\\ \sin\psi & \cos\psi & 0\\ 0 & 0 & 1 \end{array}\right]\left[\begin{array}{ccc} 1 & 0 & 0\\ 0 & \cos\theta & -\sin\theta \\ 0 & \sin\theta & \cos\theta \end{array}\right]\left[\begin{array}{ccc} \cos\gamma & 0 & \sin\gamma \\ 0 & 1 & 0\\ -\sin\gamma & 0 & \cos\gamma \end{array}\right]\\ \;\;\;\;\;=\left[\begin{array}{ccc} \cos\psi \cos\gamma -\sin\psi \sin\theta \sin\gamma & -\sin\psi \cos\theta & \cos\psi \sin\gamma +\sin\psi \sin\theta \cos\gamma \\ \sin\psi \cos\gamma +\cos\psi \sin\theta \sin\gamma & \cos\psi \cos\theta & \sin\psi \sin\gamma -\cos\psi \sin\theta \cos\gamma \\ -\cos\theta \sin\gamma & \sin\theta & \cos\theta \cos\gamma \end{array}\right] \end{array}$$

As mentioned earlier, the geographic frame “East-North-Up (E-N-U)” is selected as the navigation frame of the INS, and it is denoted as the *n*-frame. The *n*-frame is obtained by rotating the *b*-frame with an angle of γ around the *y*-axis, then θ around the *x*-axis, and next ψ around the *z*-axis.

Then, the attitude differential equation with the *n*-frame as the frame is: ${\dot{C}}_{b}^{n}=C_{b}^{n}\left(\omega_{nb}^{b}\times \right)$, where matrix $C_{b}^{n}$ represents the attitude conversion matrix from the *b*-frame to *n*-frame. The symbol $A_{BC}^{D}$, such as $\omega_{nb}^{b}$, denotes the projection of motion vector *A* of frame *C* with respect to frame *B* in frame *D*. $\omega_{nb}^{b}\times$ is an antisymmetric matrix expanded from vector $\omega_{nb}^{b}$. Since the output of the gyro is the angular velocity $\omega_{ib}^{b}$ of the b-frame with respect to the i-frame, and angular velocity vector $\omega_{nb}^{b}$ cannot be directly measured, the following transformation is required: ${\dot{C}}_{b}^{n}=C_{b}^{n}\left(\omega_{ib}^{b}\times \right)-\left(\omega_{in}^{n}\times \right)C_{b}^{n}$, where $\omega_{in}^{n}$ denotes the rotation of the *n*-frame with respect to the *i*-frame, which contains two components: the rotation of the *n*-frame caused by the earth’s rotation and the rotation of the *n*-frame due to the curvature of the earth’s surface as the INS moves near the earth’s surface. Hence, we can obtain: $\omega_{in}^{n}=\omega_{ie}^{n}+\omega_{en}^{n}$, $\omega_{ie}^{n}={\left[\begin{array}{ccc} 0 & \omega_{ie}\cos L & \omega_{ie}\sin L \end{array}\right]}^{T}$, and $\omega_{en}^{n}={\left[\begin{array}{ccc} -\frac{v_{N}^{n}}{R_{e}+h} & \frac{v_{E}^{n}}{R_{e}+h} & \frac{v_{E}^{n}}{R_{e}+h}\tan L \end{array}\right]}^{T}$, where $\omega_{ie}$ is the angular velocity of the earth’s rotation, $\omega_{ie}$ = 7.292115 × 10^−5^ rad/s, and L and h are the geographic latitude and altitude, respectively (for AUV, *h* is generally taken as a negative value, representing the depth of descent relative to the horizontal plane). In this paper, we chose a three-sample algorithm to solve the equation about ${\dot{C}}_{b}^{n}$. The following presents the specific force equation for solving the velocity and position, as shown: ${\dot{v}}_{en}^{n}=C_{b}^{n}f_{\mathrm{sf}}^{b}-\left(2\omega_{ie}^{n}+\omega_{en}^{n}\right)\times v_{en}^{n}+g^{n}$, where $f_{\mathrm{sf}}^{b}$ is the specific force measured by the accelerometer; $2\omega_{ie}^{n}\times v_{en}^{n}$ is the Coriolis acceleration generated due to the vehicle’s motion and the earth’s rotation; $\omega_{en}^{n}\times v_{en}^{n}$ is the centripetal acceleration to earth due to the vehicle’s motion; $g^{n}$ is the gravitational acceleration; $-\left(2\omega_{ie}^{n}+\omega_{en}^{n}\right)\times v_{en}^{n}+g^{n}$ is collectively denoted as the detrimental acceleration. The specific force equation about ${\dot{v}}_{en}^{n}$ shows that the geometric motion acceleration ${\dot{v}}_{en}^{n}$ of the AUV in the *n*-frame can be obtained only after the detrimental acceleration is deducted from the accelerometer output. Velocity can be obtained by integrating the acceleration once and the position once more. Therefore, the specific force equation is the basic equation for the inertial navigation solution.

The differential equations for the position (latitude, longitude, and altitude) of the INS are as follows: $\dot{L}=\frac{1}{R_{e}+h}v_{N}^{n},\dot{\;\lambda }=\frac{\sec L}{R_{e}+h}v_{E}^{n},\dot{\;h}=v_{U}^{n}$. We can rewrite them in matrix form as: $\dot{p}=M_{pv}v^{n}$, where $p=\left[\begin{array}{c} L\\ \lambda \\ h \end{array}\right]$ and $M_{pv}=\left[\begin{array}{ccc} 0 & 1/\left(R_{e}+h\right) & 0\\ \sec L/\left(R_{e}+h\right) & 0 & 0\\ 0 & 0 & 1 \end{array}\right]$. In which, $R_{e}$ is the mean radius of the earth, and value $R_{e}$ = 6,378,137 m can be taken. The above equation is the one used in the pure inertia solution.

### 2.4. Integrated Navigation Algorithm Based on CNLKF

In the following, the CNLKF model is discussed. We define the state vector **X** as follows: $X={\left[\begin{array}{ccccccccccccccc} \delta v_{E} & \delta v_{N} & \delta v_{U} & \phi_{E} & \phi_{N} & \phi_{U} & \delta \lambda & \delta L & \delta h & \varepsilon_{E} & \varepsilon_{N} & \varepsilon_{U} & \nabla_{E} & \nabla_{N} & \nabla_{U} \end{array}\right]}^{T}$. Here, $\phi_{E}$, $\phi_{N}$, and $\phi_{U}$ denote the attitude error in east, north, and up directions. $\delta v_{E}$, $\delta v_{N}$, and $\delta v_{U}$ denote the velocity error in the east, north, and up directions. δλ, δL, and δh denote the errors of longitude, latitude, and altitude. $\varepsilon_{E}$, $\varepsilon_{N}$, and $\varepsilon_{U}$ denote constant zero biases of the gyro. $\nabla_{E}$, $\nabla_{N}$, and $\nabla_{U}$ denote zero biases of the accelerometer.

The discrete LKF model of the integrated navigation system covered in this paper is shown as follows:

(6) $$\left\{\begin{array}{l} X_{k}=\Phi_{k/k-1}X_{k-1}+\Gamma_{k/k-1}W_{k-1}\\ Z_{k}=H_{k}X_{k}+V_{k} \end{array}\right.$$

$X_{k}$, $Z_{k}$, $\Phi_{k/k-1}$, $\Gamma_{k/k-1}$, and $H_{k}$ are the state vector, measurement vector, one-step transition state matrix, system noise assignment matrix, and measurement matrix, respectively. All these variables/parameters can be obtained by discretizing the time-continuous variables/parameters. Discrete period (*T*) is generally consistent with the sampling period of IMU, taken as 1–10 ms (5 ms is chosen in this article). While discretizing, the truncation term is generally taken to be 3rd or 5th order. $\Phi_{k/k-1}$ and $\Gamma_{k/k-1}$ are easy to find in textbooks for major inertial navigation. In the following CNLKF algorithm, $Z_{k}$ and $H_{k}$ are introduced. The EML is usually mounted on the head of the AUV, with its head exposed out of the AUV’s shell. The three axes of the EML form a reference frame called *em*-frame. The moving velocity of the AUV is measured by the EML in *em*-frame. The projection of the velocity vector $v_{EML}^{em}$ measured by the EML in the *n*-frame is shown as follows:

(7) $$v_{EML}^{n}=C_{b}^{n}C_{em}^{b}v_{EML}^{em}$$

where $v_{EML}^{em}$ is calculated as $v_{EML}^{em}={\left[\begin{array}{ccc} 0 & v_{EML-y}^{em} & 0 \end{array}\right]}^{T}$. $v_{EML-y}^{em}$ is measured velocity by the EML along the y-axis of the em-frame. $v_{EML}^{b}$ is calculated by $v_{EML}^{b}=C_{em}^{b}{\left[\begin{array}{ccc} 0 & v_{EML-y}^{em} & 0 \end{array}\right]}^{T}$. $C_{em}^{b}$ is the attitude transition matrix between the em-frame and b-frame. The three installation error angles between the em-frame and b-frame can be guaranteed to be small by the precision installation of EML. $C_{em}^{b}$ is approximately equal to the unit matrix. Therefore, there is a correlation: $v_{EML-y}^{b}\approx v_{EML-y}^{em}$. Each time the EML is installed, $C_{em}^{b}$ needs to be accurately calibrated by voyaging the trial to obtain installation error angles, which are compensated by Equation (7). In this paper, it is assumed that the mounting error angles are obtained by precise calibration experiments. Hence, $C_{em}^{b}$ is known. If the measurement errors are considered, the following correlations are given:

(8a) $${\tilde{v}}_{EML}^{b}+v_{C}^{b}=v^{b}+\delta v_{EML}^{b}$$

(8b) $${\tilde{v}}_{EML}^{b}=v_{ref-w}^{b}+\delta v_{EML}^{b}$$

The superscript “~” above the equations represents the measurement of a vector. $v^{b}$ and $v_{C}^{b}$ are defined in the previous Equation (2a). $v_{ref-w}^{b}$ is the real velocity reference to water of the AUV, as shown in the following equation:

(8c) $$v_{ref-w}^{b}=v^{b}-v_{C}^{b}$$

$\delta v_{EML}^{b}$ is the measurement error of the EML, which satisfies the constraint shown in Equation (8d):

(8d) $$\delta {\dot{v}}_{EML}^{b}=0$$

The *n*-frame for calculation is denoted as the *n*′-frame. If the calculation error of $C_{b}^{n}$ in Equation (7) is considered, then it is obtained that:

(9a) $${\hat{C}}_{b}^{n}=C_{b}^{n^{\prime }}=C_{n}^{n^{\prime }}C_{b}^{n}=(I-\phi \times )C_{b}^{n}$$

(9b) $$C_{n}^{n^{\prime }}\approx I-\phi \times$$

The superscript “^” in the equations indicates the calculated value. ϕ is the misalignment angle vector. With these above correlations, it is possible to derive correlations for $\delta v_{EML}^{b}$ as follows:

(10) $$\begin{array}{l} \delta v_{EML}^{n}=C_{b}^{n}C_{d}^{b}v_{ref-w}^{b}-{\hat{C}}_{b}^{n}C_{d}^{b}{\tilde{v}}_{EML}^{b}=C_{b}^{n}C_{d}^{b}v_{ref-w}^{b}-(I-\phi \times )C_{b}^{n}C_{d}^{b}(v_{ref-w}^{b}+\delta v_{EML}^{b})\\ \approx C_{b}^{n}C_{d}^{b}v_{ref-w}^{b}-C_{b}^{n}C_{d}^{b}(v_{ref-w}^{b}+\delta v_{EML}^{b})+(\phi \times )C_{b}^{n}C_{d}^{b}v_{ref-w}^{b}\\ =C_{b}^{n}C_{d}^{b}\delta v_{EML}^{b}+(\phi \times )C_{b}^{n}C_{d}^{b}v_{ref-w}^{b} \end{array}$$

The small second-order term $\left(\phi \times \right)C_{b}^{n}C_{d}^{b}v_{ref-w}^{b}$ can be neglected. Therefore, the velocity error vector caused by calculated error of $C_{b}^{n}$ can be expressed as follows:

(11) $$\delta v_{EML(Cross)}^{n}=(\phi \times )C_{b}^{n}C_{d}^{b}v_{EML}^{b}$$

The above equation shows that the velocity error contains not only the measurement error described by the measurement noise, but also the SINS misalignment angle described by the process noise and the variance of state variables. In this way, the measurement noise is coupled into process noise (as shown as follows in Equation (12)):

(12) $$\left\{\begin{array}{l} E\left[W_{k}\right]=0,E\left[W_{k}W_{j}^{T}\right]=Q_{k}\delta_{kj},\\ E[V_{k}]=0,E\left[V_{k}V_{j}^{T}\right]=R_{k}\delta_{kj},\\ E\left[W_{k}V_{j}^{T}\right]=R_{\mathrm{Cross}}\delta_{k,j-1} \end{array}\right.$$

where $\delta_{kj}$ is the Kronecker symbol defined as follows:

(13) $$\delta_{k,j-1}=\left\{\begin{array}{l} 0,k\neq j-1\\ 1,k=j-1 \end{array}\right.$$

For traditional LKF, the assumption that the measurement noise and process noise are independent of each other no longer holds. When such cross-coupling noise is obvious and it is unreasonably ignored, the accuracy of integrated navigation will be low. The prediction error of the state vector, estimation error of the state vector, and prediction error of the measurement vector are defined as $\delta {\hat{X}}_{k,k-1}=X_{k}-{\hat{X}}_{k,k-1}$, $\delta {\hat{X}}_{k}=X_{k}-{\hat{X}}_{k}$, and $\delta {\hat{Z}}_{k,k-1}=Z_{k}-{\hat{Z}}_{k,k-1}$, respectively. Subsequently, the variance and covariance can be calculated according to the following procedure. The covariance of predictions of the state vector and the measurement noise can be constructed as:

(14) $$\begin{array}{l} P_{{\hat{x}}_{k,k-1},v_{k}}=E\left[(X_{k}-{\hat{X}}_{k,k-1})V{\left(k\right)}^{T}\right]=E\left[\left(\Phi_{k,k-1}X_{k-1}+W(k-1)-\Phi_{k,k-1}{\hat{X}}_{k-1}\right)V{\left(k\right)}^{T}\right]\\ =E\left[\left(\Phi_{k,k-1}{\tilde{X}}_{k-1}+W(k-1)\right)V{\left(k\right)}^{T}\right]=E\left[W(k-1)\cdot V{\left(k\right)}^{T}\right]=R_{Cross} \end{array}$$

The variance of the prediction of the state vector can be erected as:

(15) $$P_{{\hat{x}}_{k,k-1},{\hat{x}}_{k,k-1}}=E\left[\left(X_{k}-{\hat{X}}_{k,k-1}\right){\left(X_{k}-{\hat{X}}_{k,k-1}\right)}^{T}\right]=\Phi_{k,k-1}P_{{\hat{x}}_{k-1},{\hat{x}}_{k-1}}\Phi_{k,k-1}^{T}+Q_{k-1}$$

The covariance of the prediction of the state vector and measurement vector can be set up as:

(16) $$P_{{\hat{x}}_{k,k-1},{\hat{z}}_{k,k-1}}=E\left[\left(X_{k}-{\hat{X}}_{k,k-1}\right){\left(Z_{k}-{\hat{Z}}_{k,k-1}\right)}^{T}\right]=P_{{\hat{x}}_{k,k-1},{\hat{x}}_{k,k-1}}H_{k}^{T}+R_{Cross}$$

The variance of prediction of the measurement vector is shown as:

(17) $$P_{{\hat{z}}_{k,k-1},{\hat{z}}_{k,k-1}}=E\left[\left(Z_{k}-{\hat{Z}}_{k,k-1}\right){\left(Z_{k}-{\hat{Z}}_{k,k-1}\right)}^{T}\right]=H_{k}P_{k,k-1}H_{k}^{T}+H_{k}R_{Cross}+R_{Cross}^{T}H_{k}^{T}+R_{k}$$

The gain matrix can be constructed as follows:

(18) $$K_{k}=P_{{\hat{x}}_{k,k-1},{\hat{z}}_{k,k-1}}P_{{\hat{z}}_{k,k-1},{\hat{z}}_{k,k-1}}^{-1}=(P_{{\hat{x}}_{k,k-1},{\hat{x}}_{k,k-1}}H_{k}^{T}+R_{Cross})\times {\left(H_{k}P_{k,k-1}H_{k}^{T}+H_{k}R_{Cross}+R_{Cross}^{T}H_{k}^{T}+R_{k}\right)}^{-1}$$

The variance of the state vector can be set up as:

(19) $$P_{{\hat{x}}_{k},{\hat{x}}_{k}}=E\left[\left(X_{k}-{\hat{X}}_{k}\right){\left(X_{k}-{\hat{X}}_{k}\right)}^{T}\right]=(I-K_{k}H_{k})P_{{\hat{x}}_{k,k-1},{\hat{x}}_{k,k-1}}{\left(I-K_{k}H_{k}\right)}^{-1}+K_{k}R_{k}K_{k}^{T}$$

Here, $P_{{\hat{x}}_{k,k-1},{\hat{x}}_{k,k-1}}$ and $P_{{\hat{x}}_{k},{\hat{x}}_{k}}$ are similar to $P_{k,k-1}$ and $P_{k}$ in standard LKF. Equations (15)–(19) constitute LKF for handling cross-coupling noise. In this filter, ${\hat{X}}_{k,k-1}$ and ${\hat{X}}_{k-1}$ are calculated in the same way as conventional LKF. The difference between CNLKF (as shown by Equations (15)–(19)) and standard LKF shows that the introduced cross-coupling noise will affect $P_{{\hat{x}}_{k,k-1},{\hat{z}}_{k,k-1}}$, $P_{{\hat{z}}_{k,k-1},{\hat{z}}_{k,k-1}}$ and $K_{k}$, but it has no effect on $P_{{\hat{x}}_{k,k-1},{\hat{x}}_{k,k-1}}$ and $P_{{\hat{x}}_{k},{\hat{x}}_{k}}$. In conventional LKF, $K_{k}$ determines the estimation accuracy of ${\hat{X}}_{k,k-1}$ and $Z_{k}$, while ${\hat{X}}_{k,k-1}$ and $Z_{k}$ are used to estimate ${\hat{X}}_{k}$. In Equation (18), cross-noise term $R_{Cross}$ appears in both the numerator and denominator. In conventional LKF, the noise term $R_{k}$ appears only in the denominator, and the effect of $R_{Cross}$ is not considered. The method shown in Equation (18) is in principle more reasonable and more accurate.

Based on the state vector $X_{k}$ and the measurement vector $Z_{k}$ described above, the cross-noise term $R_{Cross}$ in Equation (12) is a non-negative definite matrix whose dimension is 15 × 3. As described in this section, the attitude misalignment angle will result in an error of the projected velocity. The expression of $R_{Cross}$ is:

(20a) RCross=RCross11000RCross22000RCross33 012×3 

where $R_{Cross11}$, $R_{Cross22}$, and $R_{Cross33}$ are cross-coupling values. In the following, the setting of initial values of the CNLKF is discussed. Based on the factory report of the inertial sensor in our experiment, the zero bias of the gyro (accelerometer) is 0.008°/h (20 μg). The initial position of the AUV is 110.42296° longitude and 21.23709° latitude. In general, the velocity measurement error of the EML is less than 0.1 m/s. The measurement noise of the EML set in this algorithm is (0.1 m/s)^2^, and the cross-coupling value is (0.02 m/s)^2^. Based on experience, the initial values and parameters of the CNLKF are set as shown in Equation (20b).

(20b) X0=000000000000000T P0=diag[0.25 m2·s−20.25 m2·s−20.25 m2·s−27.62×10−3 rad7.62×10−3 rad3.048×10−2 rad9.85×10−10 rad9.85×10−10 rad1 m22.12×10−10 rad·s−22.12×10−10 rad·s−22.12×10−10 rad·s−28.64×10−6 m2·s−48.64×10−6 m2·s−48.64×10−6 m2·s−4] Qk=Q0=diag[3.84×10−8 m2·s−43.84×10−8 m2·s−43.84×10−8 m2·s−41.95×10−8 rad1.95×10−8 rad1.95×10−8 rad2.28×10−11 rad2.28×10−11 rad0.01 m2000000] Rk=R0=diag0.01 m2·s−20.01 m2·s−20.01 m2·s−2RCross=4.0×10−4 m2·s−20004.0×10−4 m2·s−20004.0×10−4 m2·s−2 012×3 

Since this algorithm dose not employ adaptive LKF and does not consider variations of **R***_k_* and **Q***_k_* during recursion process, it is assumed that **R***_k_* = **R**_0_, **Q***_k_* = **Q**_0_.

While the AUV is sailing underwater, it receives only the measured velocity from the EML. The INS performs CNLKF (FOF), and it works in the integrated mode of INS/HMM (or INS/EML). The observation vector in such a mode is as follows:

(21) $$Z_{k}=\left[\begin{array}{c} v_{INS-E}^{n}-{v^{\prime }}_{HMM-E}^{n}\\ v_{INS-N}^{n}-{v^{\prime }}_{HMM-N}^{n}\\ v_{INS-U}^{n}-{v^{\prime }}_{HMM-U}^{n} \end{array}\right]$$

where ${v^{\prime }}_{HMM}^{n}$ is calculated as follows: ${v^{\prime }}_{HMM}^{n}=v_{HMM}^{n}+v_{C}^{n}$. The specific calculation process for the ocean current velocity $v_{C}^{n}$ is presented in the previous Figure 1. The ocean current velocity is obtained by subtracting the velocity vector output by the MALKF from the velocity vector output by the HMM after coordinate transformation, and $v_{HMM}^{n}$ is calculated as follows: $v_{HMM}^{n}=C_{b}^{n}\cdot {\left[\begin{array}{ccc} 0 & v_{HMM-y}^{b} & 0 \end{array}\right]}^{T}$. ${v^{\prime }}_{HMM}^{n}$ contains three components: ${v^{\prime }}_{HMM}^{n}={\left[\begin{array}{ccc} {v^{\prime }}_{HMM-E}^{n} & {v^{\prime }}_{HMM-N}^{n} & {v^{\prime }}_{HMM-U}^{n} \end{array}\right]}^{T}$. The measurement matrix is as follows:

(22) $$H_{k}=\left[\begin{array}{ccccc} \begin{array}{ccc} 1 & 0 & 0\\ 0 & 1 & 0\\ 0 & 0 & 1 \end{array} & 0_{3\times 3} & 0_{3\times 3} & 0_{3\times 3} & 0_{3\times 3} \end{array}\right]$$

Before performing the first GNSS-based calibration, Equations (21) and (22) are used as the observation equations for integrated navigation. The above equations in this section are the model and calculation formula of CNLKF.

Therefore, for the parameters **X**_0_, **P**_0_, **Q**_k_, and **R**_k_ of conventional LKF, the setting of initial values is basically the same as Equation (20b), except that $R_{Cross}$ is replaced by the following expression:

(23) $$R_{Cross}=\left[0_{15\times 3}\right]$$

### 2.5. MALKF-Based GNSS-Based Calibration Algorithm

In traditional LKF, the fixed system noise and measurement noise variance matrix is generally used. The large/small measurement noise represents low/high reliability of measurement. When the AUV is on the water surface, the stability of the GNSS signal is poor due to the influence of waves, wind, and currents. Occasionally, the measurement noise changes dramatically. In this case, the conventional processing method of fixed measurement noise signifies a large error of integrated navigation. The actual GNSS noise is much larger than the setting constant value of **R**_0_. To ensure the accuracy of integrated navigation, the measurement noise is required to be automatically adjusted during filtering based on real-time measurement. In this paper, an innovative MALKF is employed for GNSS-based calibration to solve the problems of the unstable GNSS signal and the outliers. The algorithm estimates the measurement noise in real time based on an innovation sequence. The innovation sequence is defined as:

(24) $$\varepsilon_{k}=Z_{k}-H_{k}X_{k,k-1}=H_{k}(X_{k}-X_{k,k-1})+V_{k}$$

The KF is optimal choice when innovation sequence is zero-mean Gaussian white noise. Taking the variance on both sides of the above equation, it is obtained that:

(25) $$E[\varepsilon_{k}\varepsilon_{k}^{T}]=H_{k}P_{k,k-1}H_{k}^{T}+R_{k}$$

Thus, the measurement noise ***R****_k_* can be obtained with the innovation sequence, as shown by Equation (26).

(26) $$R_{k}={\varepsilon_{k}\varepsilon_{k}^{T}-H}_{k}P_{k,k-1}H_{k}^{T}$$

Only elements on the diagonal are retained in the calculation. In practice, a moving data window is usually employed online to estimate the measurement noise, and the average of the previous *m* moments of measurement noise is set as the current noise:

(27) $$R_{k}=\frac{1}{m}\sum_{i=0}^{m-1}R_{k-i}$$

**Figure 3:**
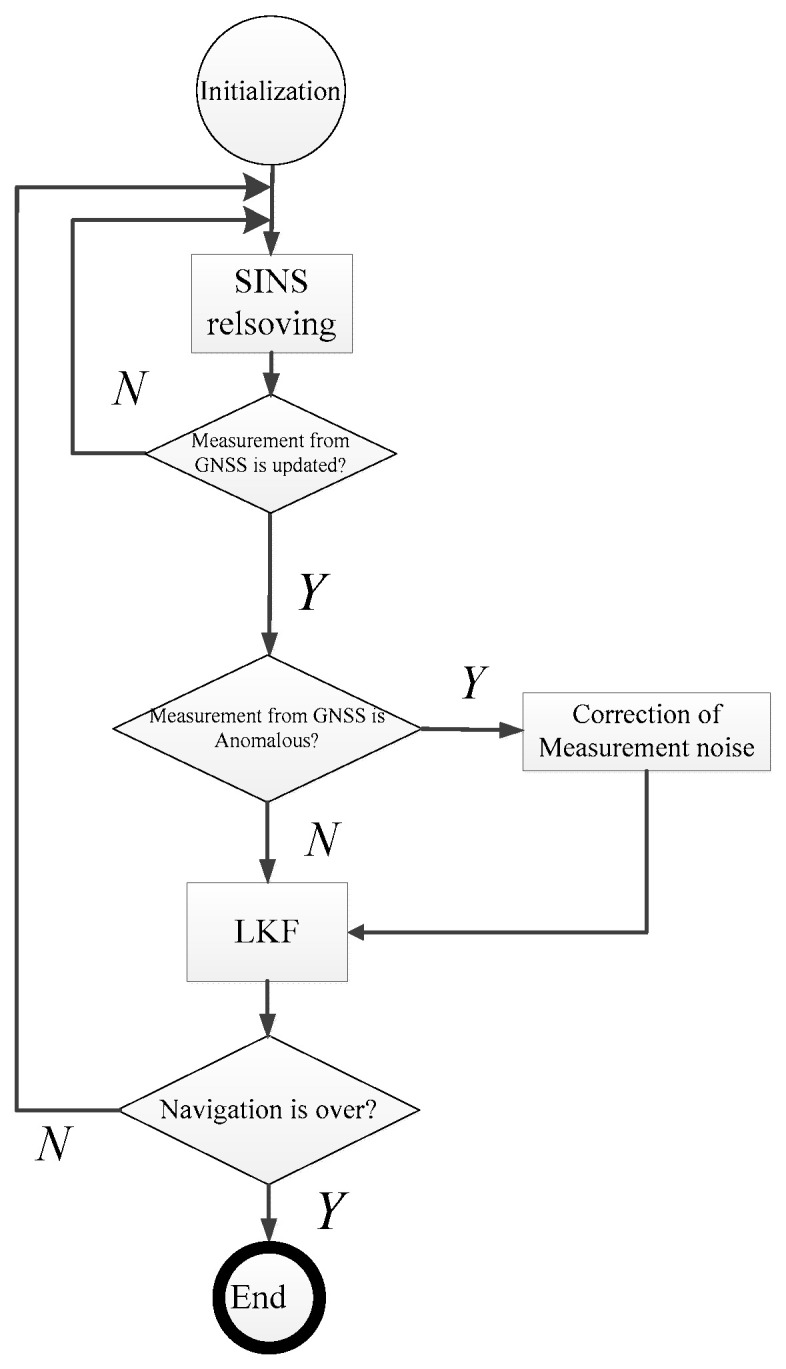
Flow chart of MALKF.

In the above equation, *m* is the length of the moving average window. If GNSS’s measurement is abnormal, it switches to be MALKF described above. The overall algorithm process of this filtering is shown in Figure 3. When GNSS data are normal, the INS works in the mode of an ordinary LKF and uses the moving data window to estimate measurement noise in real time. When the GNSS data are judged to be abnormal by *χ*^2^-test, it switches to the adaptive filtering mode to reduce the impact of outliers from the GNSS on the accuracy of integrated navigation.

Figure 3 shows the detailed flow chart of MALKF. As shown in the figure, the adaptive LKF operation is performed when the GNSS measurement is anomalous, and the conventional LKF operation is conducted when the GNSS measurement is normal. It can greatly improve the stability and robustness of KF during GNSS-based calibration. Even the outlier from GNSS does not cause a large positioning error or large estimation error of current speed. In order to ensure the stability of KF, it switches to the adaptive LKF only when an abnormal GNSS measurement is reliably identified by the *χ*^2^-test.

(28) $$A_{k}=H_{k}P_{k,k-1}H_{k}^{T}+R_{k}$$

The parameter $\gamma_{k}$ is introduced to describe whether the GNSS measurement is an outlier, as shown in Equation (29a):

(29a) $$\gamma_{k}=\varepsilon_{k}^{T}{A_{k}^{-1}\varepsilon }_{k}$$

where $\gamma_{k}$ satisfies $\chi^{2}$-distribution, as shown in the following equation:

(29b) $$\left\{\begin{array}{l} {\mathrm{abnormal}:\gamma }_{k}\geq T_{D};\\ {\mathrm{normal}:\gamma }_{k}<T_{D} \end{array}\right.$$

The parameter *T_D_* can be set according to the rate of false alarm.

When the AUV on the water surface can receive both measurements from the GNSS (position) and EML (velocity), INS performs MALKF (SOF). In this case, the INS works in the integrated mode of INS/GNSS/EML (or INS/GNSS/HMM). The observation vector in such a mode is as follows:

(30) $$Z_{k}=\left[\begin{array}{c} v_{INS-E}^{n}-{v^{\prime }}_{HMM-E}^{n}\\ v_{INS-N}^{n}-{v^{\prime }}_{HMM-N}^{n}\\ v_{INS-U}^{n}-{v^{\prime }}_{HMM-U}^{n}\\ \lambda_{INS}-\lambda_{GNSS}\\ L_{INS}-L_{GNSS}\\ h_{INS}-h_{DG} \end{array}\right]$$

(31) $$H_{k}=\left[\begin{array}{ccccc} \begin{array}{ccc} 1 & 0 & 0\\ 0 & 1 & 0\\ 0 & 0 & 1 \end{array} & 0_{3\times 3} & 0_{3\times 3} & 0_{3\times 3} & 0_{3\times 3}\\ 0_{3\times 3} & 0_{3\times 3} & \begin{array}{ccc} 1 & 0 & 0\\ 0 & 1 & 0\\ 0 & 0 & 1 \end{array} & 0_{3\times 3} & 0_{3\times 3} \end{array}\right]$$

where ${v^{\prime n}}_{HMM-i}\left(i=E,N,U\right)$ and $v_{INS-i}^{n}\left(i=E,N,U\right)$ are the three velocity components in the *n*-frame output from HMM and INS, respectively. The vector ${v^{\prime n}}_{HMM}$ has three components ${v^{\prime n}}_{HMM-i}$, which are obtained by multiplying the vector ${\left[\begin{array}{ccc} 0 & {v^{\prime b}}_{HMM-y} & 0 \end{array}\right]}^{T}$ by the matrix $C_{b}^{n}$. $\lambda_{GNSS}$, $L_{GNSS}$, and $h_{DG}$ are the longitude and latitude from GNSS, and the depth from DG, respectively. $\lambda_{\mathrm{INS}}$, $L_{INS}$, and $h_{INS}$ are longitude, latitude, and depth calculated by INS. $R_{k}$ and $R_{Cross}$ corresponding to Equations (30) and (31) are:

(32a) $$R_{k}=diag\left[\begin{array}{cccccc} 0.01\;m^{2}\cdot s^{-2} & 0.01\;m^{2}\cdot s^{-2} & 0.01\;m^{2}\cdot s^{-2} & 9\;m^{2} & 9\;m^{2} & 9\;m^{2} \end{array}\right]$$

(32b) RCross=4.0×10−4 m2·s−2    4.0×10−4 m2·s−2 03×12  4.0×10−4 m2·s−2  03×15  

The rest of the initial values of MALKF are consistent with those in Equation (20b).

**Figure 4:**
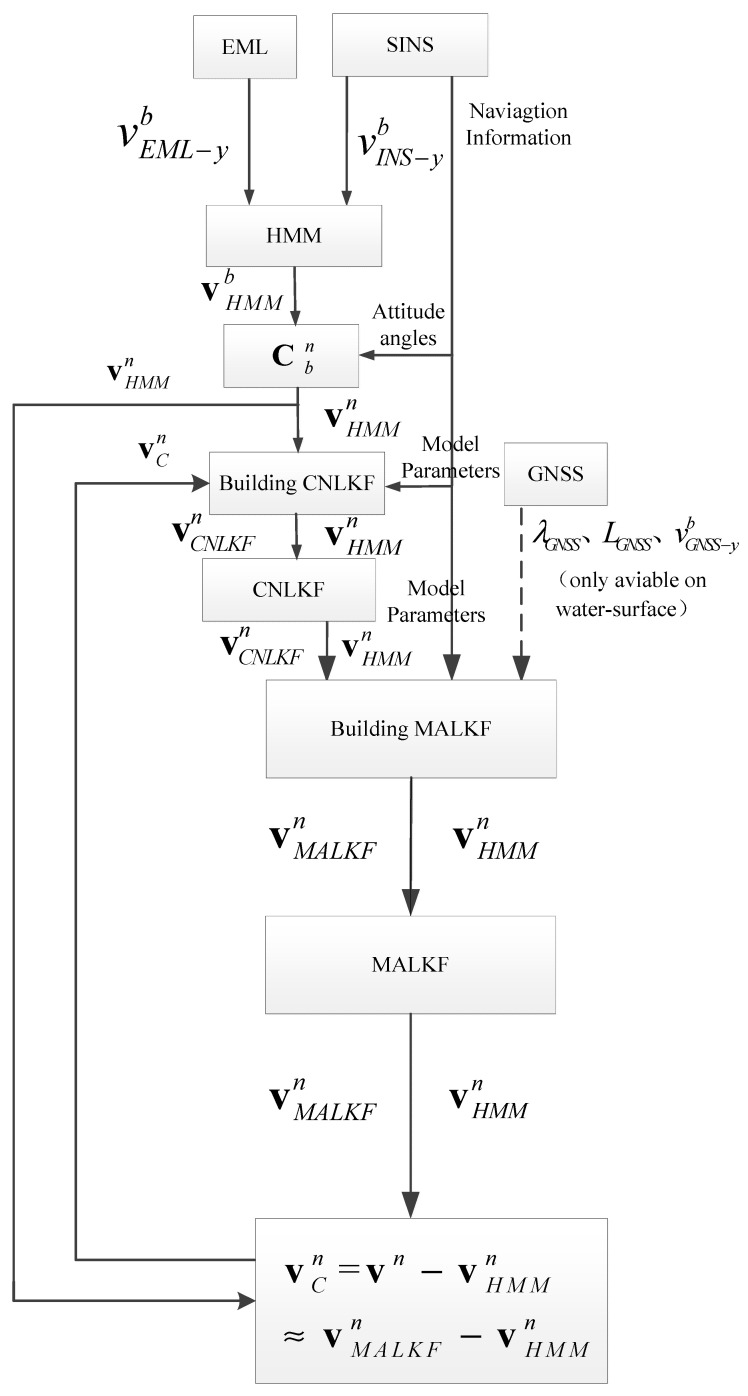
Simplified flow chart of the proposed algorithm.

Figure 4 shows a simplified flow chart of MALKF. This process is the core part of the complete process shown in Figure 1. In Figure 4, $v_{MALKF}^{n}\approx v^{n}$. This means that the output of MALKF is regarded as the real velocity vector in the *n*-frame.

### 2.6. Feedback Correction Algorithm

The accuracy of velocity and position from pure inertial reckoning can be improved by substituting the ground velocity from the SOF into the Specific Force Equation (10) and related elements of the LKF model. This modified LKF model can be applied in CNLKF or MALKF. The accuracy of the attitude angle from the pure inertial solution can be improved by substituting $\delta \omega_{ie}^{n}$ and $\delta \omega_{en}^{n}$ into the attitude angle updating process and related elements of LKF. $\delta \omega_{ie}^{n}$ and $\delta \omega_{en}^{n}$ can be calculated by Equation (33).

(33) $$\left\{\begin{array}{l} \delta \omega_{ie}^{n}={\left[\begin{array}{ccc} 0 & \omega_{ie}\cos(\delta L) & \omega_{ie}\sin(\delta L) \end{array}\right]}^{T}\\ \delta \omega_{en}^{n}={\left[\begin{array}{ccc} -\frac{v_{HMM-N}^{\prime }}{R_{Mh}}+\frac{v_{INS-N}}{R_{Mh}} & \frac{v_{HMM-E}^{\prime }}{R_{Mh}}-\frac{v_{INS-E}}{R_{Mh}} & \frac{v_{HMM-E}^{\prime }}{R_{Nh}}\tan(L+\delta L)-\frac{v_{SINS-E}}{R_{Nh}}\tan(\delta L) \end{array}\right]}^{T} \end{array}\right.$$

Here, latitude *L* is from second-order LKF. Highly accurate pure inertial solution implies stable and fast converge of the CNLKF/MALKF recursion.

**Figure 5:**
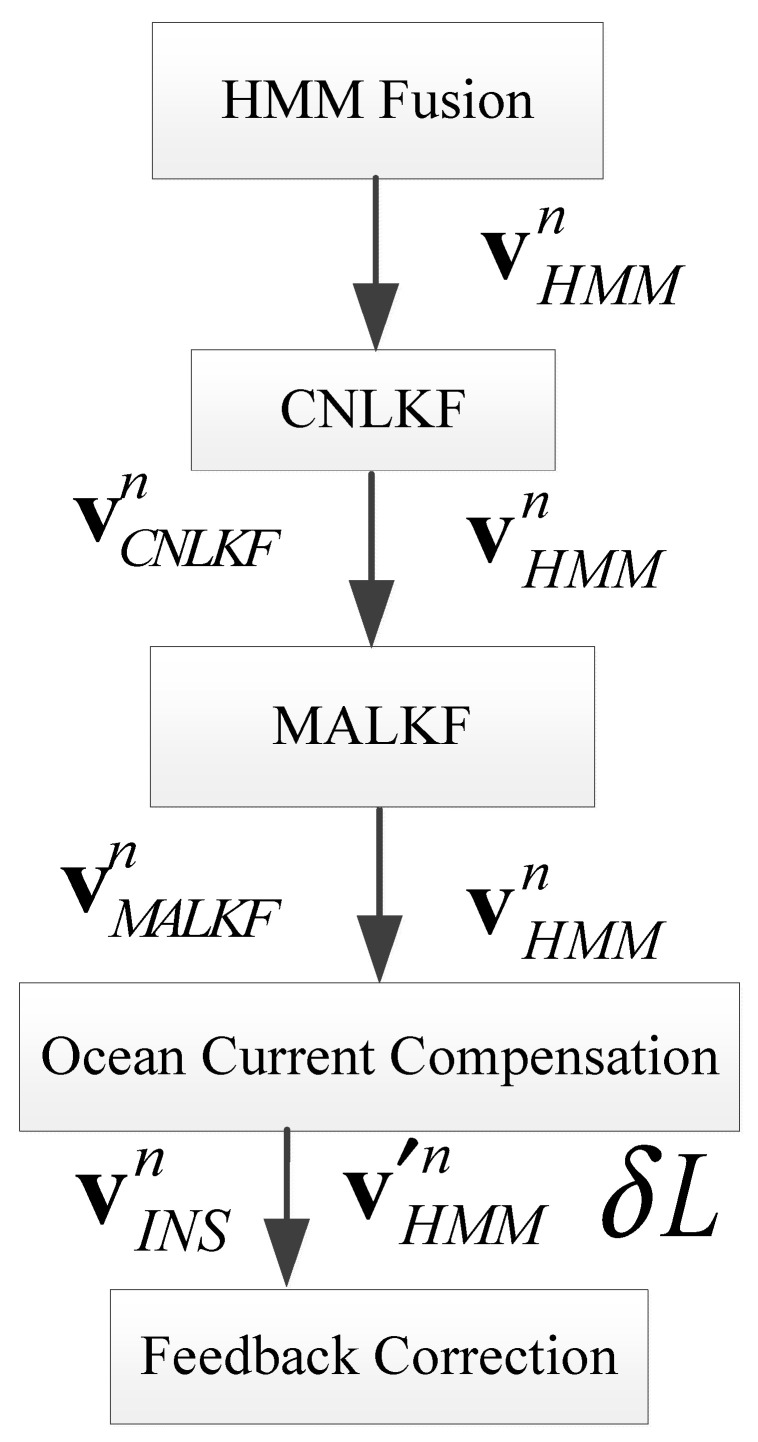
The five core modules of the proposed algorithm.

The above Section 2.2, Section 2.3, Section 2.4, Section 2.5 and Section 2.6 constitute the complete calculation process of this algorithm. The detailed process is as follows: After receiving forward velocity from the INS and velocity through water from the EML, we fuse the velocities by HMM. The denoised velocity through water can be obtained. By inputting the denoised velocity through water into the first-order KF, the navigation parameters can be obtained. After receiving the position from the GNSS, we input the position and results from the FOF into the SOF, and the navigation parameters of the SOF can be obtained. The current velocity can be estimated by subtracting ground velocity by the SOF from velocity through water. By feeding the current velocity back to the HMM to compensate the velocity through water, the compensated velocity (ground velocity) can be obtained. The compensated velocity, which is defined as velocity compensated by the estimated current speed, is approximately equal to the ground speed. The navigation parameters from SOF and compensated velocity are employed for feedback correction to obtain highly accurate navigation parameters. The five core modules of the detailed process (shown in Figure 1 above) are given in Figure 5.

## 3. Experimental and Simulation Test

**Figure 6:**
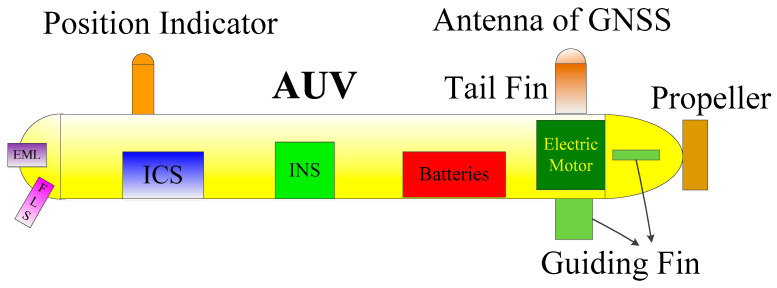
Diagram of the AUV layout.

**Figure 7:**
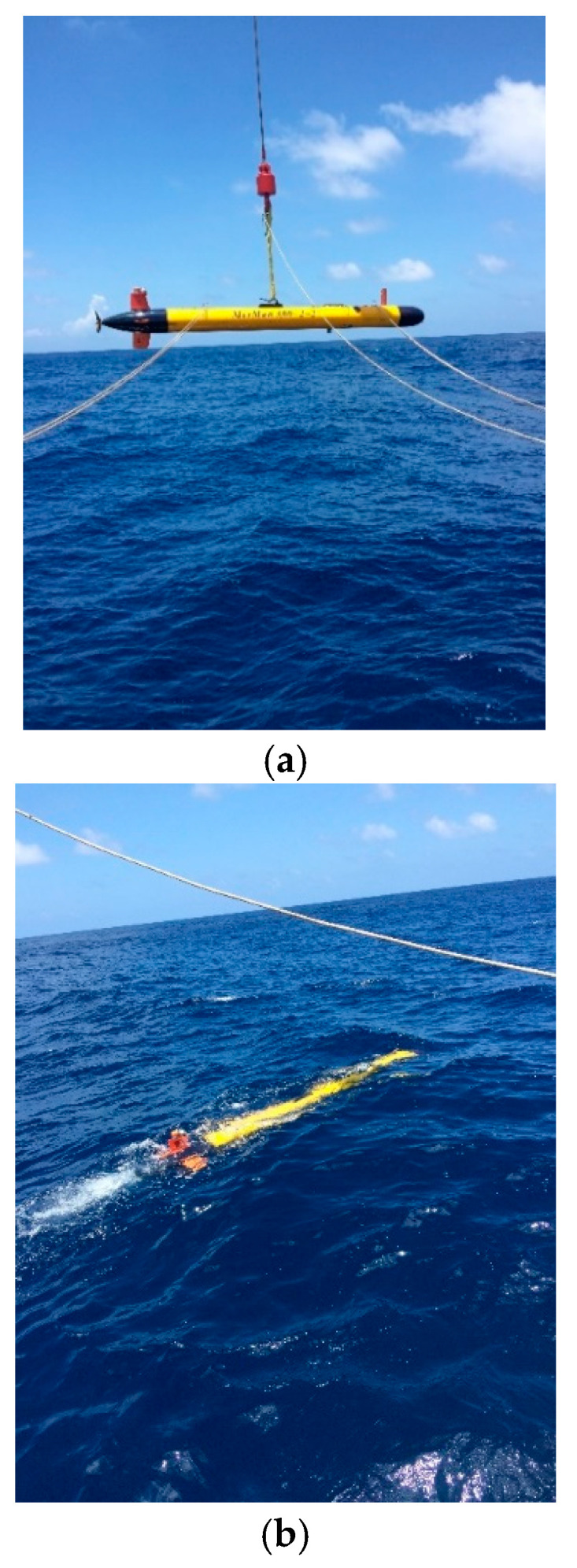
(**a**) Photograph of deployment (from ship to water surface) of the AUV. (**b**) Photograph of the AUV sailing on the water surface.

**Figure 8:**
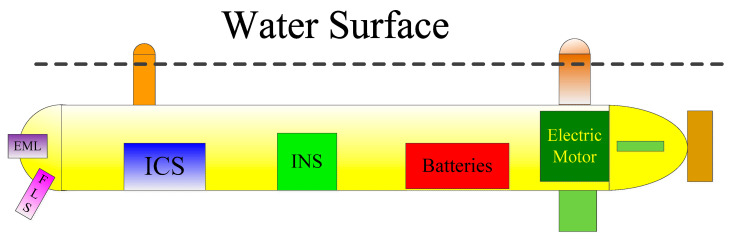
Schematic diagram of the AUV voyaging on the water surface.

The algorithm is verified by conducting a sea trial of the AUV in the sea. In this experiment, a small-sized tail rotor propelled AUV is employed and its layout is shown in Figure 6, where ICS and FLS are the integrated control system and forward-looking sonar, respectively. The head of the AUV is equipped with EML and FLS for velocity measurement and obstacle avoidance, respectively, and ICS is located in the anterior half segment of the AUV. In order to avoid the virtual linear velocity caused by the lever arm effect and the AUV’s rotation, the INS is installed at the rotation center of the AUV. Virtual linear velocity induces navigation effect. The battery is located in the posterior half part of the AUV, and the propulsion motor is located in the rear section. A propeller is installed at the last end of the AUV. A guidance rudder plate is installed on the abdomen of the rear part of the AUV, and two guidance rudder plates are installed on the two sides of the AUV’s rear section (only one is shown in Figure 6). A fin stabilizer is mounted on the top of the rear section, and a GNSS receiver (antenna and circuit board) is mounted on top of the fin stabilizer. A position-indicating light is mounted on top of the AUV’s front section. The navigation system mainly consists of the EML, the fiber optic gyro (FOG)-based INS, and the GNSS. The GNSS consists of an antenna and circuit board. The circuit board is installed below the GNSS antenna in the receiver. The zero biases and gyro random walks of accelerometers in the INS are described in the previous Section 2.4. The velocity measurement error of this EML is less than ±0.1 kn (1 kn = 1.852 km/h) or ±1% *V* (*V* is the actual speed of the AUV, and the larger of two errors is taken). The positioning error of GNSS is ≤3 m, and the velocity measurement error of the GNSS is ≤0.2 m/s. Photos of the testing field are shown in Figure 7a,b. Figure 7a shows the deployment of the AUV. Figure 7b is a photograph of the AUV sailing on the water surface. In order to preserve the accurate positions of the sampling points on the AUV’s trajectory as a reference, the AUV voyages on the water surface throughout the test so that GNSS signals can be received in real time, as shown in Figure 8, and the GNSS antenna of the AUV is always above the water surface while the AUV is moving. In order to study the accuracy of the INS/EML-integrated mode, GNSS measurements are not applied in this mode, and they are only used as benchmarks to calculate the positioning errors. Only when GNSS-based calibration is conducted, GNSS information is input into MALKF as a measurement, and then the integrated navigation system is in the INS/GNSS/EML mode. The frequency of GNSS-based calibration is once every 25 min. When GNSS-based calibration is performed, GNSS measurement is input into MALKF for calibration. Due to the powerful calculation capability of the navigation circuit board, the moment that a valid GNSS measurement is received is the moment that calibration begins, and the time spent by one calibration is in the order of ms. The first GNSS-based calibration is performed at the start-point of the trajectory. In this experiment, the data update periods of the gyro and accelerometer of INS, EML, and GNSS are 5 ms, 1 s, and 1 s. Therefore, the period of pure inertial solution is 5 ms. The time update period of CNLKF/MALKF is 5 ms. The measurement update periods of the EML and GNSS are 1 s and 25 min.

Figure 7a shows a photograph of the test site of the deployment of the AUV. The crane on the working mother ship is employed to lift and put the AUV on the water’s surface, and the crane ring is located near the center of the AUV. Figure 7b shows a photo of the AUV voyaging on the water surface. As analyzed above, the GNSS antenna is kept above the water surface during the sailing.

Figure 8 shows a schematic diagram of the AUV moving on the surface of the water. In the experiment, the depth of the AUV is precisely controlled, keeping the AUV’s position-indicating light and GNSS antenna above the water surface. Compared with the AUV’s main body, the position indicating light, fins, and GNSS antenna are small, and they are far away from the EML. Hence, their disturbances to the water current near the EML can be negligible during the experiment. The current velocity estimated by the EML’s measurement and proposed algorithm is the velocity of the current near the water surface. Therefore, the measurements obtained from buoys can be used as benchmarks to calculate the error of estimating current speed. When the AUV is moving with high maneuverability or high speed or impacted by strong wind/wave, the GNSS antenna will be below water surface, and some outliers of the GNSS measurement may appear. Therefore, we utilize MALKF, which is more advanced than conventional LKF, for the fusion of the GNSS measurement.

**Figure 9:**
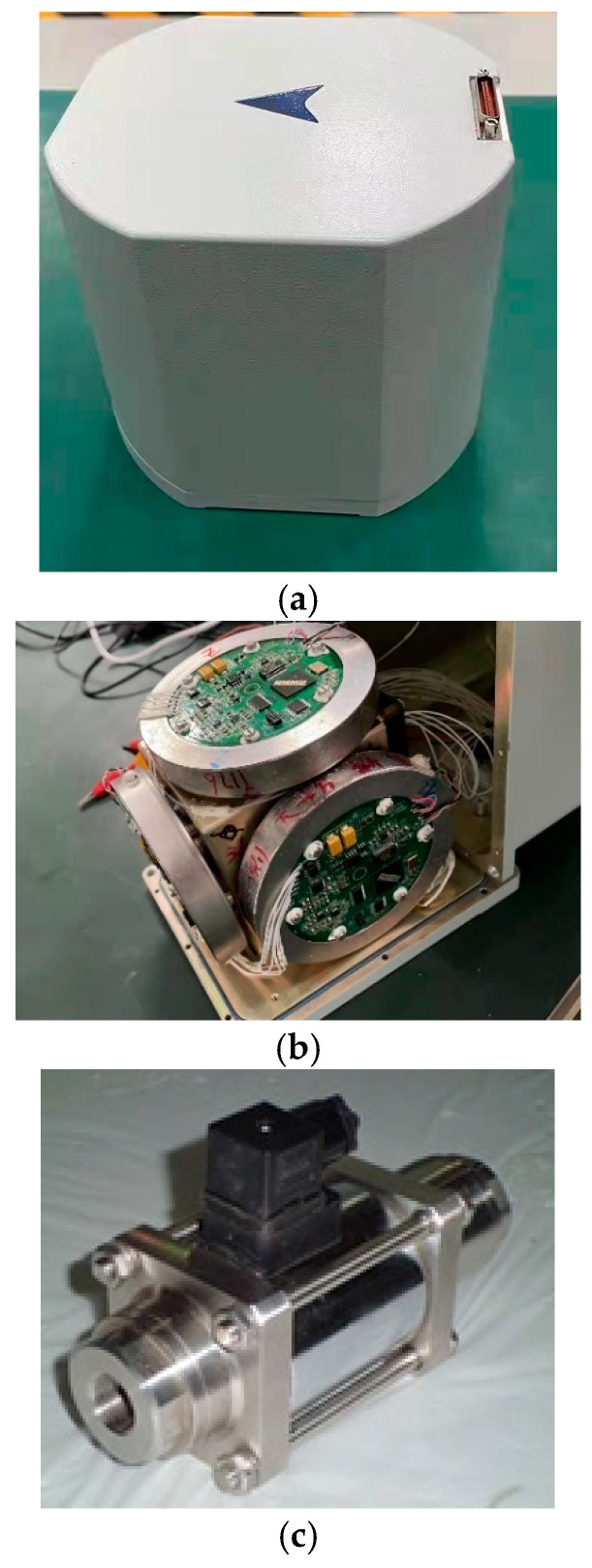
(**a**) Physical photos of the INS. (**b**) Physical photos of the internal structure of the INS. (**c**) Physical photos of the EML.

Physical photos of the shell of the INS, the internal structure of the INS, and the EML are shown in Figure 9a–c. The FOG-based INS is selected. The arrow in Figure 9a points to the heading of the AUV. The INS contains three FOGs, three quartz flexible accelerometers, and the navigation circuit board. Figure 9b shows the three orthogonally mounted gyros in the IMU. The EML (shown in Figure 9c) is mounted at the center hole of the AUV’s front cover. The velocity measurement of the EML is input into the INS for integrated navigation.

The experimental process is introduced as follows. After the AUV is placed on the water surface, the INS begins to align. After the alignment is completed, a calibration by GNSS is performed, and the AUV begins its sea trial. The point of GNSS-based calibration is the starting point of this trajectory. The AUV voyages for about 3825 s. Then, the AUV selects a new starting point and performs GNSS-based calibration to start a new voyage. The above processes are repeated, and a total of six voyages are conducted. This article selects the first of six voyagesfor analysis and discussion.

**Figure 10:**
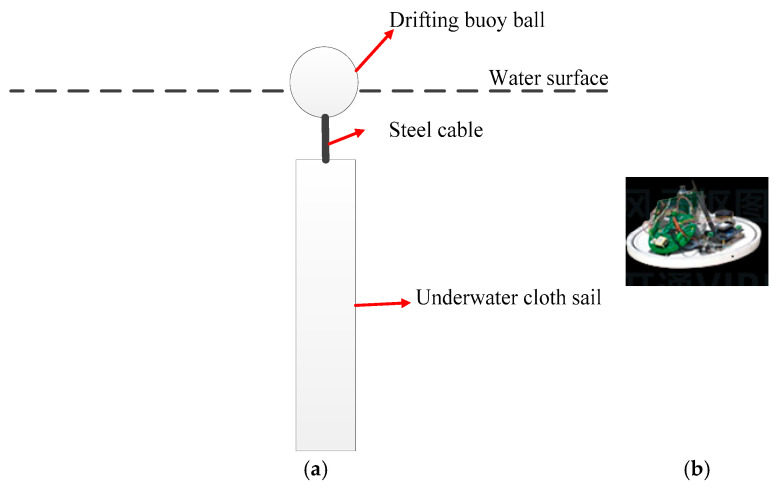
(**a**) Schematic diagram of the drifting buoy. (**b**) Physical picture of devices inside the buoy ball.

As analyzed above, in order to obtain accurate current speed as a benchmark, we utilize buoys for the accurate measurements of speed of current of water layer near the surface. Figure 10a shows a schematic diagram of a buoy structure. The buoy contains a drifting float above the water surface, an underwater fabric sail, and a steel cable connecting the float and fabric sail. The underwater fabric sail is designed to improve the attitude stability of the float. All the mass, shape, volume, and fluid properties are optimized to ensure the attitude stability of the float without dragging the float (ensuring that the speed of float is equal to that of the surface current). The underwater sail consists of a halyard, four traction ropes, and a main body, whose material is nylon. The photo shown in Figure 10b shows instruments and sensors inside a floating ball, including: the Iridium communication module, battery pack, the GNSS module (Beidou/GPS 2-in-1), the temperature sensor, and the controller. The measurements of temperature and position/velocity by GNSS are transmitted back to the server by the Iridium communication system, with a data integrity rate ≥ 98% and GNSS update period of 1 s. From these data, the speed and direction of the current and temperature of the water surface can be deduced. The velocity error of GNSS inside the buoy is ≤0.1 m/s, and the positioning error is ≤2 m. Therefore, the current speed measured by the buoy can be considered accurate. In this test, 13 buoys are placed near the trajectory of AUV at approximately 1 km intervals for measurement. Accurate current speed is obtained from real-time measurement of the closest buoy to the AUV. The buoys are put into the water by the mother ship. Since the depth of the AUV’s central axis is approximately 400 m, the variation in current velocity with depth is generally very slow. Our extensive current velocity testing in this water body indicates that the velocity difference between the surface currents and the currents at the depth of the AUV’s central axis typically does not exceed 0.03 m/s. Hence, the current speed measured by the buoy is almost equal to that of water layer where the AUV is located.

**Figure 11:**
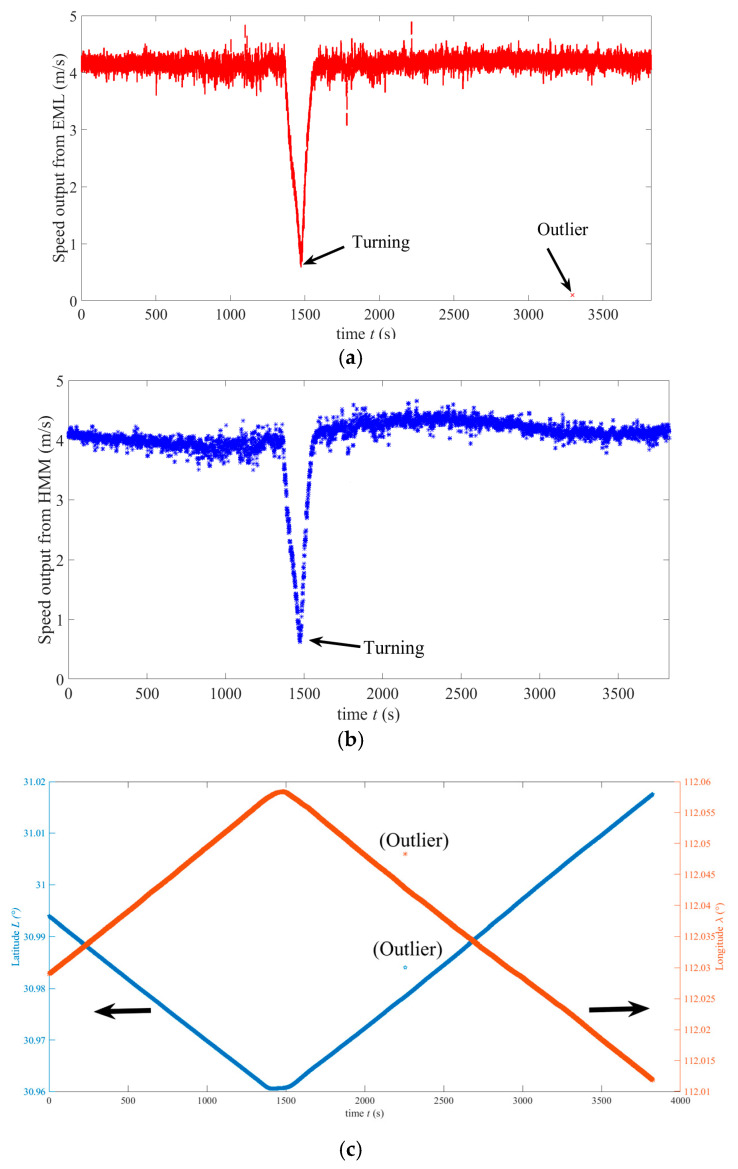
(**a**) Speed measured by the EML (including one outlier). (**b**) Speed after HMM filtering. (**c**) Position measured by the GNSS (with one outlier).

Figure 11a–c shows the velocity measured by EML, the velocity after HMM processing, the position (longitude and latitude) measured by GNSS, and the local zoom of the measured position during the whole cruise, respectively. During the sailing of the AUV, the input power and rotational speed of propulsion motor remain stable. Hence, average velocity through the water of the AUV is stable. This is evident in Figure 11a. From Figure 11a, it can also be seen that the velocity measured by EML is noisy and has one outlier (At 3318 s, the corresponding velocity is 0.02 m/s, which is far from true value). Without HMM pre-processing, the outlier will cause a large positioning error of the INS/EML-integrated navigation. From this figure, it can be seen that the average speed is about 4.05 m/s, and the minimum speed at the turning is as low as 0.55 m/s. The velocity after HMM filtering shown in Figure 11b shows that the noise has been greatly suppressed and outliers have been effectively removed. The weak fluctuation of the mean velocity in this figure is caused by the error of velocity by pure inertial calculation. From Figure 11c, it can be seen that there is one outlier in the measured latitude and longitude. There is an outlier at 2258 s, with an error of several hundred meters in both longitude and latitude measurements.

**Figure 12:**
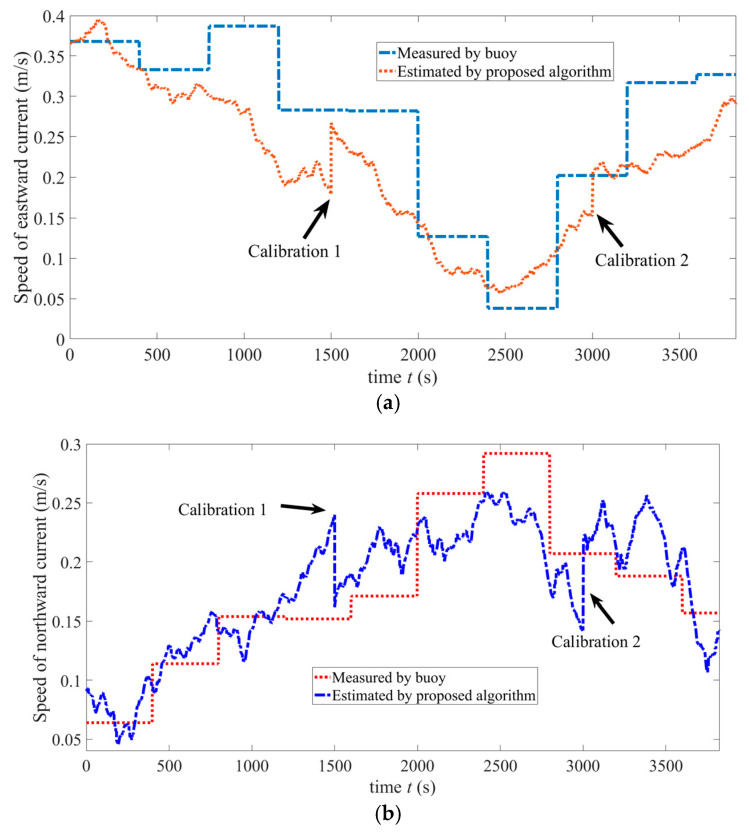
(**a**) Estimated and actual speeds of the eastward current. (**b**) Estimated and actual speeds of the northward current.

Figure 12a shows the speed of the eastward current estimated by the proposed algorithm and that measured by a buoy. Here, the *n*-frame is used to describe water current. While moving, two GNSS calibrations at 1500 s and 3000 s are identified by arrows. As seen in this figure, the maximum estimation error appears at the moment of 1211 s, which is 0.14 m/s, and the estimation error of the endpoint is 0.041 m/s. Figure 12b shows the speed of the northward current estimated by the proposed algorithm and that measured by a buoy. This figure shows that the maximum estimation error at the moment of 2573 s, which is 0.095 m/s, and the estimation error of the endpoint is 0.032 m/s. From these two figures, it can be seen that water current flows in the northeast direction. Due to a GNSS calibration conducted at the starting point, the estimation errors of eastward and northward currents at the start are relatively small. These results indicate that the proposed algorithm can effectively estimate the velocities of ocean currents.

In order to present the estimated and measured current speeds more clearly, some data in Figure 12a,b is tabulated and shown in Table 1 below. The time interval for the tabulation is 12 min. It should be noted that the frequency of GNSS-based calibration is once every 25 min, so the estimation error of the current speed is small after a GNSS calibration.

**Figure 13:**
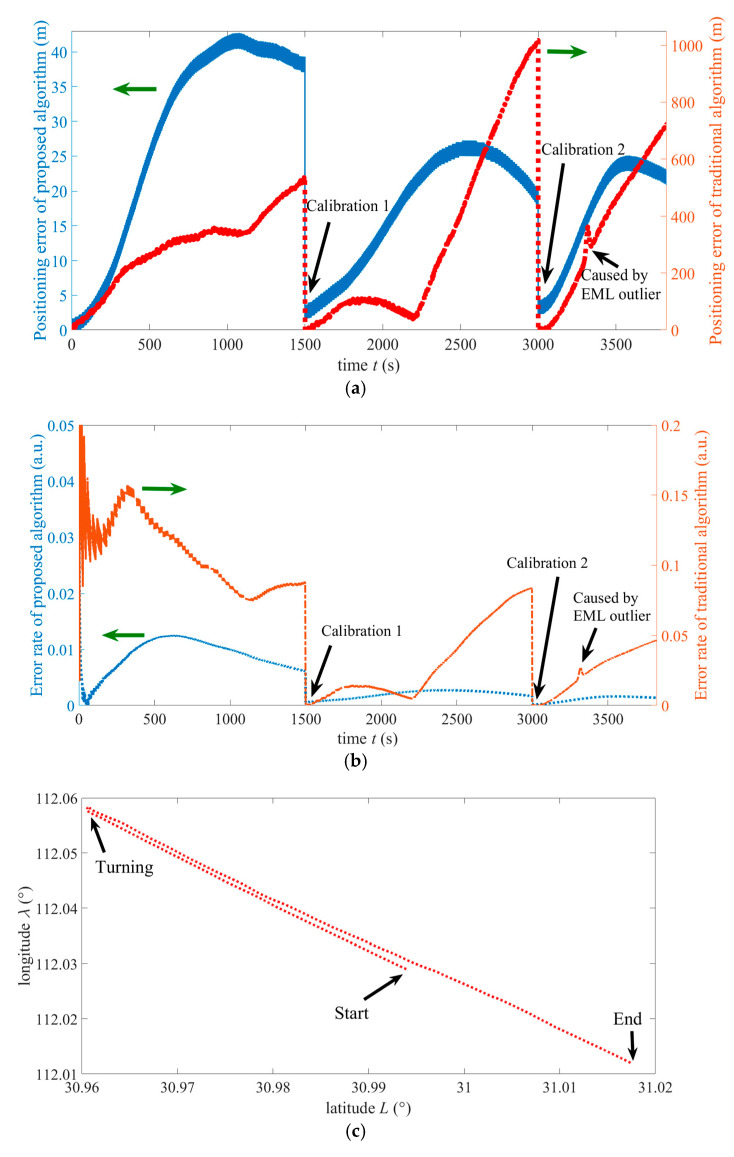

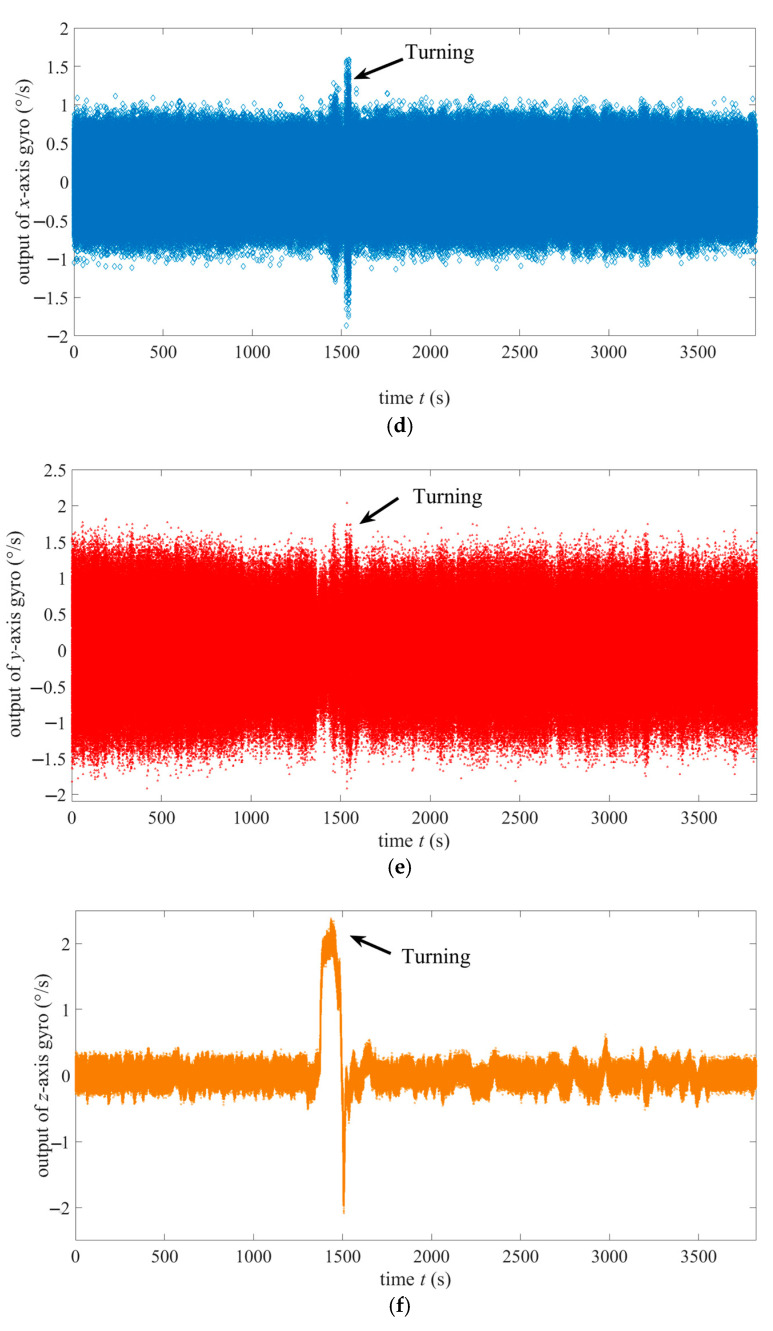

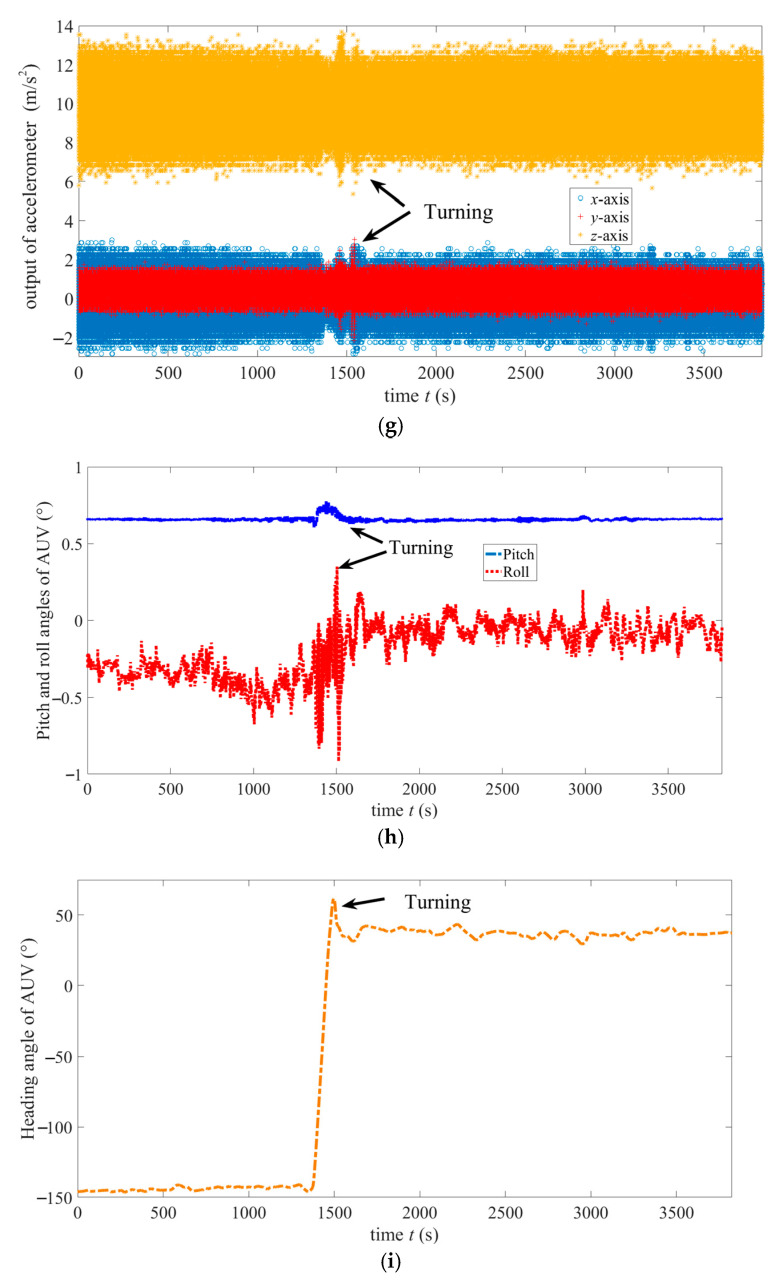
(**a**) Positioning error of the proposed algorithm and conventional LKF. (**b**) Positioning error rate of the proposed algorithm and conventional LKF. (**c**) Trajectory based on GNSS measuring positions. (**d**) Output of *x*-axis gyros in IMU. (**e**) Output of *y*-axis gyros in IMU. (**f**) Output of *z*-axis gyros in IMU. (**g**) Output of three accelerometers. (**h**) Pitch and roll angles of the AUV. (**i**) Heading angle of the AUV.

Figure 13a shows the positioning errors of the proposed algorithm and conventional LKF. The positioning error is obtained by taking the square root of sum of squares of latitude and longitude positioning errors. Here, the GNSS measurement update period of conventional LKF is also set to be 25 min. The benchmark datum used for calculation is the position measured by GNSS. In this figure, the small peak at 3318 s is caused by the measurement outlier of the EML. From this figure, the maximum positioning error of the proposed (conventional) algorithm occurs at the moment of 1005 s (2999 s) with an error of 41.1 m (1008.2 m). Figure 13b shows the error rates of the proposed algorithm and conventional LKF. The error rate is defined as the current positioning error divided by the current voyaging distance. The maximum error rate of the proposed (conventional) algorithm occurs at the moment of 508 s (413 s) with an error of 1.2% (15.1%). When analyzing the maximum error rate, few initial inaccurate points are discarded (due to the very small voyaging distance, the error rate is unreasonable and should be abandoned). The positioning error and error rate at the endpoint of the proposed (conventional) algorithm are 20.5 m (712.1 m) and 0.134% (4.64%), respectively. Every error or error rate of the proposed algorithm is significantly reduced compared to the conventional algorithm. It illustrates the superiority of this algorithm. In particular, the positioning error of the INS/EML mode of the conventional method increases sharply when an outlier in the EML output appears, while this error of proposed method does not increase sharply due to the HMM pre-processing technique. Figure 13c shows the trajectory based on GNSS measuring positions (actual trajectory). This trajectory includes two approximately straight line segments and one turning segment. Figure 13a–f shows the outputs of three gyroscopes (*x*-axis, *y*-axis, and *z*-axis). From these figures, it can be seen that the output of the *z*-axis gyro is only relatively large when the AUV is turning. Figure 13g shows the outputs of three accelerometers. Due to the stable motion of the AUV, outputs of all three accelerometers are small. The maximum absolute values of the *x*-axis and *y*-axis accelerometers are less than 3.1 m/s^2^ and 3.5 m/s^2^, respectively. Figure 13h shows pitch and roll angles of the AUV. Figure 13i shows the heading angle of the AUV. From these two figures, it can be seen that there is a significant variation in heading angle only when the AUV is turning.

**Figure 14:**
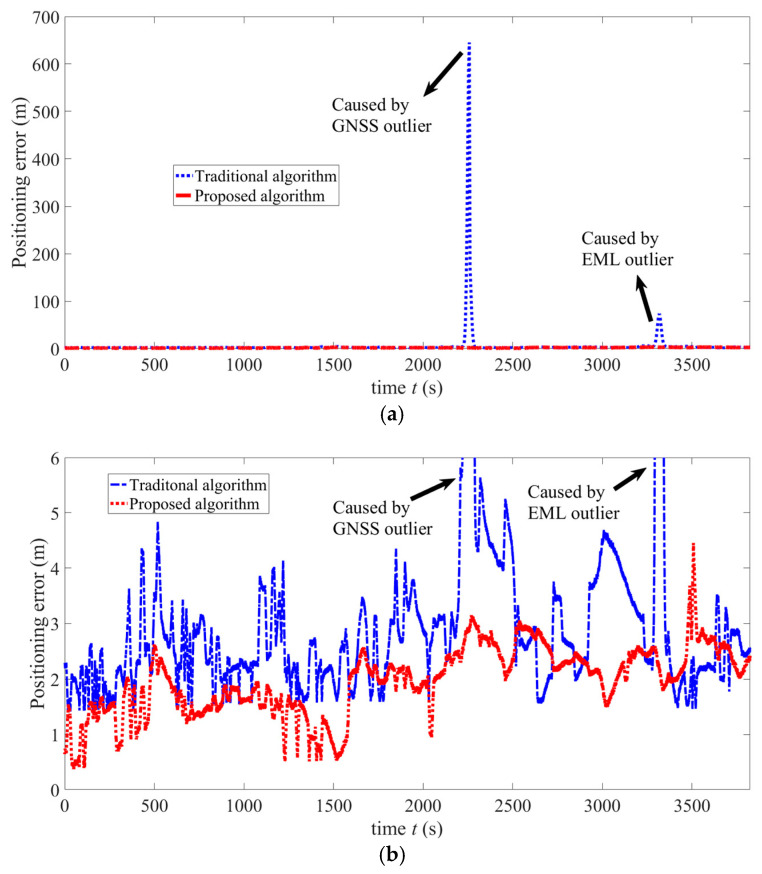

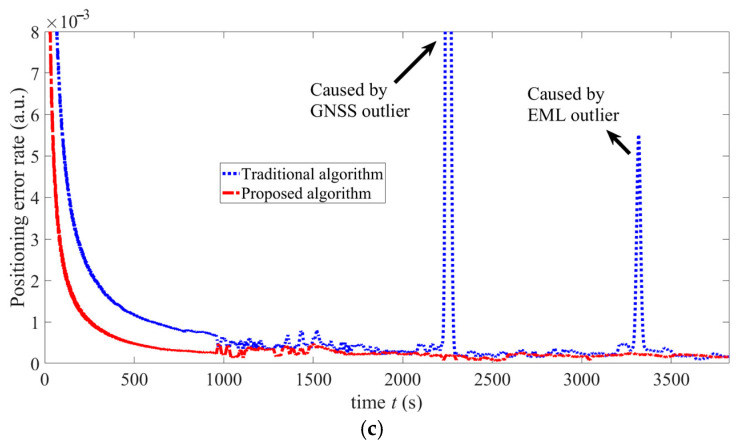
Simulation results of the GNSS with a measurement update period of 1 s. (**a**) Figure of positioning errors of the proposed and conventional algorithms. (**b**) Local figure (except two peaks caused by two outliers) of positioning errors of the proposed and conventional algorithms. (**c**) Local figure (except peak caused by GNSS outlier) of the positioning error rates of the proposed algorithm and conventional algorithm.

The probability of outliers appearing in GNSS measurements is very low. While the measurement update period of MALKF is 25 min, positions from GNSS are all normal values. The measurement is shown in the above Equation (30). Hence, advantages of MALKF cannot be manifested. In order to specifically verify the excellent robustness of MALKF, we perform another simulation with a measurement update period of 1 s. This period encounters a case in which the outlier of the GNSS measurement appears. By setting the measurement update period of MALKF to be 1 s, results are obtained and shown in Figure 14a,b. Figure 14a shows that the positioning error of the conventional algorithm increases sharply whenever the outlier of the EML or GNSS appears, while the error of the proposed algorithm does not increase sharply at any outlier point. The maximum error is 651 m. Because GNSS is frequently utilized to calibrate, the positioning error of the proposed algorithm is of meter level. Figure 14b shows a local zone (parts of curves except for GNSS and EML outliers) in Figure 14a. As shown by Figure 14c, the characteristics of error rates are similar to those of error. The maximum error rate is 7.1%.

The results in Figure 14a–c shows that the proposed algorithm can indeed effectively solve a sharp increase of error of MALKF caused by a measurement outlier.

To thoroughly evaluate the performance of the proposed algorithm, we compared it with traditional algorithms, as well as the UKF (Unscented Kalman Filter) and CKF (Cubature Kalman Filter) algorithms. The computational results are presented in Table 2 below. Using offline simulation techniques, we applied the four algorithms listed in Table 2 to process the experimental data shown in Figure 13c–i. The UKF algorithm utilizes the UKF to replace the CNLKF and MALKF in Figure 1, while the CKF algorithm uses the CKF to replace the CNLKF and MALKF. To ensure a fair comparison, both the UKF and CKF algorithms include HMM preprocessing and outlier removal and replacement for the GNSS data.

The results from the table indicate that the maximum positioning error and the maximum ocean current estimation error of the UKF and CKF algorithms outperform the proposed algorithm. This is because the proposed algorithm is based on the LKF framework, and its performance still lags behind that of more advanced nonlinear filters. However, the proposed algorithm demonstrates a computational time advantage. Using the ATmega256 AVR microcontroller as the computing chip, the computation times for the proposed algorithm, UKF, and CKF are approximately 1.8, 3.5, and 3.8 times that of the traditional algorithm, respectively. Therefore, the proposed algorithm has a relatively low computational time and holds certain engineering application value.

## 4. Conclusions

In summary, the INS/EML and INS/EML/GNSS-integrated navigation algorithm for small-sized AUVs is proposed in this paper. The algorithm employs techniques including HMM pre-processing, CNLKF, MALKF, GNSS measurement-based estimation of current speed, and feedback correction of LKF.

The results of the AUV’s sea trial show that the positioning error and error rate at the endpoint of the proposed algorithm are 20.5 m and 0.134%, respectively, when the measurement update period of MALKF is set to be 25 min. The error and error rate of conventional algorithm at the endpoint are 712.1 m and 4.64%. The maximum estimation error of the eastward (northward) current speed appears at the moment of 1211 s (2573 s), which is 0.14 m/s (0.095 m/s), and the estimation error at the endpoint is 0.041 m/s (0.032 m/s).

These results show that the algorithm in this paper has advantages of high accuracy, strong anti-interference ability, and wide application range. Its engineering practicality is high, and it is expected to be applied to small AUVs, ROVs, underwater gliders, and other marine equipment.

## Figures and Tables

**Table 1:** Estimated and measured speeds of eastward and northward currents.

Time (s)	Measured Eastward Current (m/s)	Estimated Eastward Current (m/s)	Measured Northward Current (m/s)	Estimated Northward Current (m/s)
0	0.370	0.369	0.062	0.093
720	0.335	0.312	0.117	0.132
1440	0.293	0.225	0.151	0.192
2160	0.132	0.105	0.251	0.206
2880	0.21	0.162	0.201	0.193
3600	0.331	0.265	0.155	0.143

**Table 2:** Comparison of results of different filtering algorithms.

Method	Maximum Positioning Error (m)	Error in Estimation of Maximum Current Speed (m/s)
Proposed	40.8	0.095
Traditional	1015.4	——
UKF	32.4	0.083
CKF	29.7	0.071

## Data Availability

The data presented in this study are available on request from the corresponding author.
